# Ru(ii)–Re(i) binuclear photocatalysts connected by –CH_2_XCH_2_– (X = O, S, CH_2_) for CO_2_ reduction†
†Electronic supplementary information (ESI) available: Stern–Volmer plots of the dyad; UV-vis spectra of the OERs of **Ru** and **Re**; UV-vis spectral changes during the photocatalytic reaction; ESI-MS spectrum of the SEC peak at 36.1 min after the 120 min photocatalytic reaction using **Ru(CH_2_OCH_2_)Re**; IR spectral change by the formation of **Ru(CH_2_OCH_2_)Re(CO)_3_(X)** (X = ^–^OC_2_H_4_N(C_2_H_4_OH)_2_ or ^–^OC(O)OC_2_H_4_N(C_2_H_4_OH)_2_); Stern–Volmer plots for **Ru(CH_2_OCH_2_)Re(CO)_3_(X)** (X = ^–^OC(O)OC_2_H_4_N(C_2_H_4_OH)_2_); changes of SEC chromatograms, ESI-MS spectra, and IR spectra during the photocatalytic reaction using **Ru(CH_2_CH_2_CH_2_)Re**; changes of a ESI-MS spectrum after the photocatalytic reaction; ESI-MS spectra of the SEC peak at 36.5 min and 37.8 min after the 120 min photocatalytic reaction using **Ru(CH_2_SCH_2_)Re**. See DOI: 10.1039/c4sc03710c


**DOI:** 10.1039/c4sc03710c

**Published:** 2015-03-02

**Authors:** Eishiro Kato, Hiroyuki Takeda, Kazuhide Koike, Kei Ohkubo, Osamu Ishitani

**Affiliations:** a Department of Chemistry , Graduate School of Science and Engineering , Tokyo Institute of Technology , 2-12-1-NE1, O-okayama , Meguro-ku , Tokyo 152-8550 , Japan . Email: ishitani@chem.titech.ac.jp; b CREST , Japan Science and Technology Agency , 4-1-8 Honcho , Kawaguchi-shi , Saitama 322-0012 , Japan; c National Institute of Advanced Industrial Science and Technology , 16-1 Onogawa , Tsukuba , Ibaraki 305-8569 , Japan

## Abstract

We developed Ru(ii)–Re(i) supramolecular photocatalysts in which each metal complex unit is connected by a –CH_2_XCH_2_– (X = O, S, CH_2_) chain. The photocatalyst with X = O exhibited the best photocatalytic efficiency for CO_2_ reduction in the reported systems using a NAD(P)H model compound as an electron donor because the introduced oxygen atom strengthened the oxidation power of the **Ru** photosensitizer unit in the excited state and accelerated electron transfer from the one-electron-reduced **Ru** photosensitizer unit to the **Re** catalyst unit. In contrast, the catalytic ability of the photocatalyst with X = S rapidly decreased during irradiation because the supramolecular structure split into mononuclear complexes. A detailed mechanism for the efficient photocatalytic reaction involving these supramolecular photocatalysts was investigated for the first time.

## Abstract 2

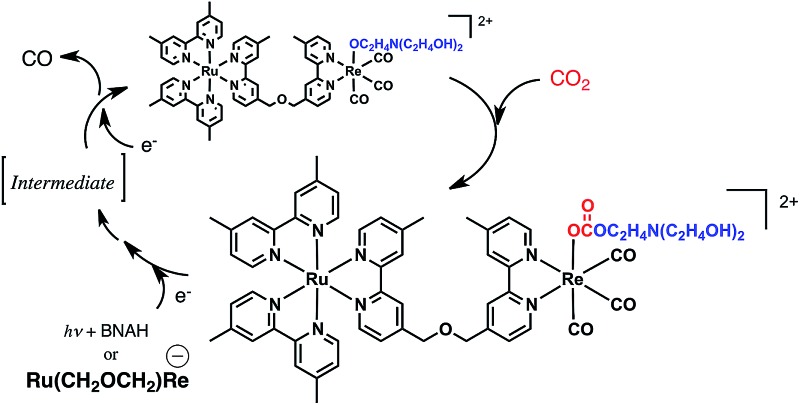
New Ru(ii)–Re(i) diads with bridging ligands constructed of two diimines connected by –CH_2_OCH_2_– or –CH_2_SCH_2_– were synthesized and investigated as photocatalysts with enhanced oxidation power.

## Introduction

The conversion of CO_2_ into high-energy compounds using solar energy is a potential technology for addressing both the problem of global warming and the shortage of fossil fuel resources.^1^ Some transition metal complexes can broadly absorb visible light and function as redox photosensitizers, which drive one-electron-transfer reactions.^2^ However, because the reduction of CO_2_ to stable compounds requires multi-electron insertion into CO_2_, a catalyst is also required for photocatalytic CO_2_ reduction systems.^3^ Electrochemical catalysts for CO_2_ reduction using transition metal complexes^5^ or a pyridinium cation^6^ have been developed, and some of them have been applied for photocatalytic systems with redox photosensitizers.^7^ As typical examples, mixed systems consisting of [Ru(N⁁N)_3_]^2+^ (N⁁N = diimine ligand) as a photosensitizer and Co, Ni, and Ru complexes as a catalyst have been reported to photocatalyze CO_2_ reduction to CO and/or HCOOH using visible light.^4^

**Chart 1:**
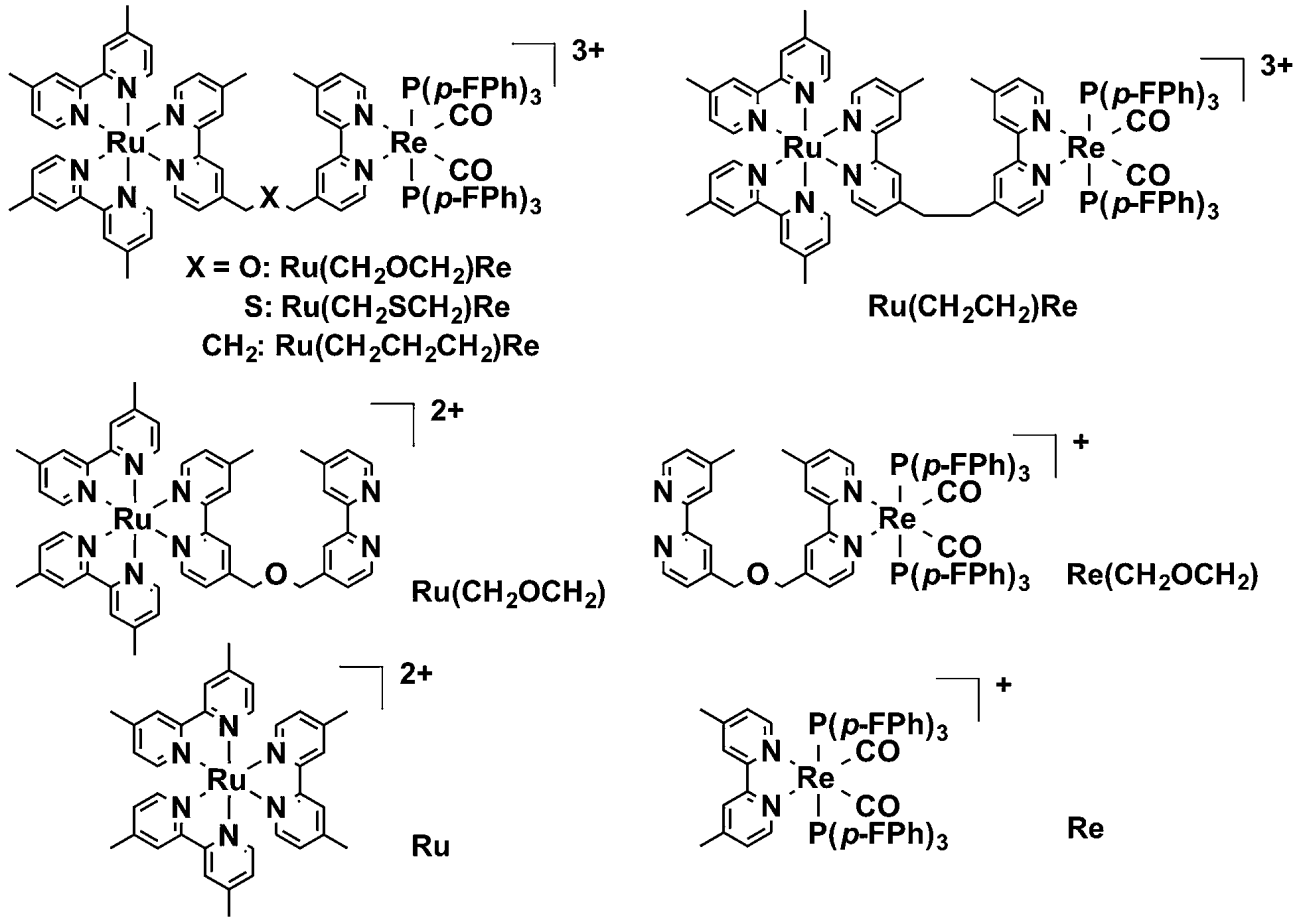
Structures and abbreviations of the metal complexes.

We have developed supramolecular photocatalysts with a Ru(ii) photosensitizer and a Re(i) catalyst connected to each other *via* a bridging ligand consisting of two diimine moieties connected with an alkyl chain,^8^*e.g.*, **Ru(CH_2_CH_2_)Re** (Chart 1): the quantum yield (*Φ*_CO_) and the turnover number (TON_CO_) of CO formation were 0.15 and 207, respectively, by using 1-benzyl-1,4-dihydronicotinamide (BNAH) as a reductant,8*e* which has been the most efficient supramolecular photocatalyst for CO_2_ reduction of those reported.8*b* The efficiency and durability of these diad photocatalysts are much higher than those of the corresponding mixed systems. However, these systems still have a problem; the low oxidation power of the excited Ru unit of the diads severely limits the kinds of reductants that can be used. For example, only 62% of the excited Ru units of **Ru(CH_2_CH_2_)Re** were reductively quenched by a relatively high concentration (0.1 M) of BNAH, which has been often used as a sacrificial electron donor. This result means that 38% of the absorbed photons are wasted in the first process of the photocatalytic reaction and cannot be used for CO_2_ reduction. Therefore, improvement of the oxidation power of the excited Ru unit is required to enhance the photocatalytic activity of the diad. One method for improving the oxidation ability of the excited Ru unit has been reported: introducing electron-withdrawing groups, *e.g.*, –CF_3_, into the peripheral ligands.8*a* Although greater efficiency of the quench by a reductant could be achieved, the photocatalytic ability of the diad with such a Ru unit for CO_2_ reduction was much lower than that of the corresponding diad without the electron-withdrawing groups in the Ru unit. Electron transfer from the reduced Ru unit to the Re unit could not efficiently proceed because the added electron was localized in the peripheral ligands with the electron-withdrawing groups and the redox potential of the Ru unit became more positive than that of the Re unit. Improving the photocatalytic activity of the supramolecular photocatalyst therefore requires both strengthening the oxidation power of the photosensitizer unit and achieving efficient electron transfer from the reduced photosensitizer unit to the catalyst unit.

Recently, we also reported a new hybrid CO_2_ reduction photocatalyst in which a supramolecular photocatalyst with phosphate anchor groups was adsorbed onto the semiconductor photocatalyst TaON.^9^ This hybrid photocatalyst exhibited both strong oxidation power and selective reduction power for CO_2_ reduction because of the Z-scheme mechanism, in which stepwise two-photon excitation can make both a hole as a powerful oxidant in the valence band of TaON and an electron in the photosensitizer unit of the diad. One of the most important keys to improving the efficiency of this promising hybrid photocatalyst is the reconciliation of the stronger oxidation power of the excited photosensitizer unit and the efficient intramolecular electron transfer in the diad because efficient interfacial electron transfer should proceed from the conduction band of TaON to the excited photosensitizer unit of the diad and then to the catalyst unit during the photocatalytic reduction of CO_2_.

In this report, new Ru(ii)–Re(i) diads with bridging ligands constructed of two diimines connected by –CH_2_OCH_2_– or –CH_2_SCH_2_– were synthesized and investigated as photocatalysts with enhanced oxidation power; their enhanced oxidation power stems from the greater electronegativity of the bridging ligands' oxygen and sulfur atoms compared to that of the central carbon atom in the alkyl chains such as –CH_2_CH_2_CH_2_– (Chart 1). The diad with the –CH_2_OCH_2_– chain exhibited the best performance as a supramolecular catalyst for CO_2_ reduction using BNAH as the reductant. A detailed mechanism of CO_2_ reduction using the Ru–Re diads is also reported for the first time.

## Results and discussion

The diads were synthesized by the following method: the reaction of [(dmb)_2_RuCl_2_]^2+^ (dmb = 4,4′-dimethyl-2,2′-bipyridine) and the bridging ligand gave **Ru(CH_2_XCH_2_)** (X = O, S, CH_2_) with a bridging ligand with a non-coordinated diimine moiety; **Ru(CH_2_XCH_2_)** was subsequently reacted with [Re(CO)_3_{P(*p*-FPh)_3_}_2_(OTf)] to give **Ru(CH_2_XCH_2_)Re** in 8–32% yield calculated on the basis of the amount of [(dmb)_2_RuCl_2_]^2+^ used.

**Fig. 1:**
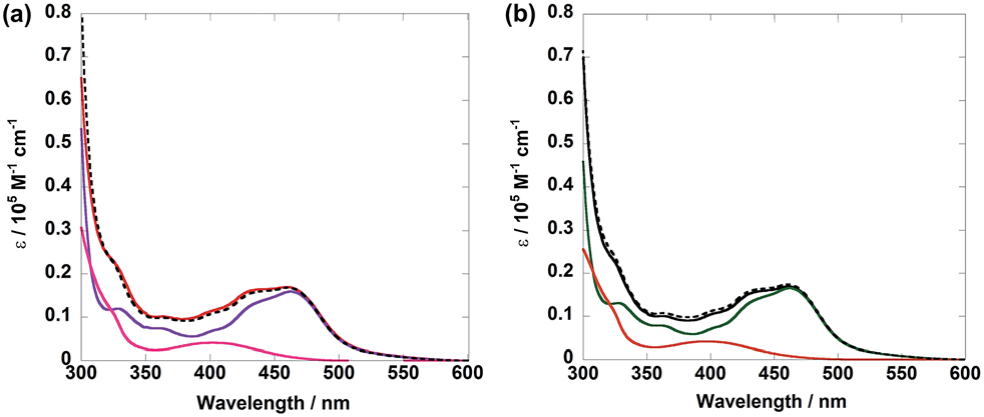
UV-vis absorption spectra of (a) **Ru(CH_2_OCH_2_)Re** (red), **Ru(CH_2_OCH_2_)** (purple), and **Re(CH_2_OCH_2_)** (pink), and (b) **Ru(CH_2_CH_2_CH_2_)Re** (black), **Ru** (green), and **Re** (red). The dotted lines show the 1 : 1 summation spectrum of (a) **Ru(CH_2_OCH_2_)** and **Re(CH_2_OCH_2_)** and (b) **Ru** and **Re**. Dimethylformamide (DMF) was used as the solvent.

Fig. 1a shows the UV-vis absorption spectra of **Ru(CH_2_OCH_2_)Re** and its model mononuclear complexes (**Ru(CH_2_OCH_2_)** and **Re(CH_2_OCH_2_)**), and Fig. 1b shows those of **Ru(CH_2_CH_2_CH_2_)Re** and its models (**Ru** and **Re**). The spectra of both diads are approximately consistent with the summation spectra of their model complexes, as shown by the dotted lines in the figures. The results indicate that no strong electronic interaction occurs between the Ru and Re units in the ground states of both diads. The broad absorption at 400–550 nm is assignable to metal-to-ligand charge-transfer (^1^MLCT) absorption of the Ru unit, whereas that at 350–460 nm is attributable to ^1^MLCT absorption of both Ru and Re units. The strong absorption band at approximately 300 nm is attributed to π–π* absorption of the diimine ligands of both the Ru and the Re units.

**Fig. 2:**
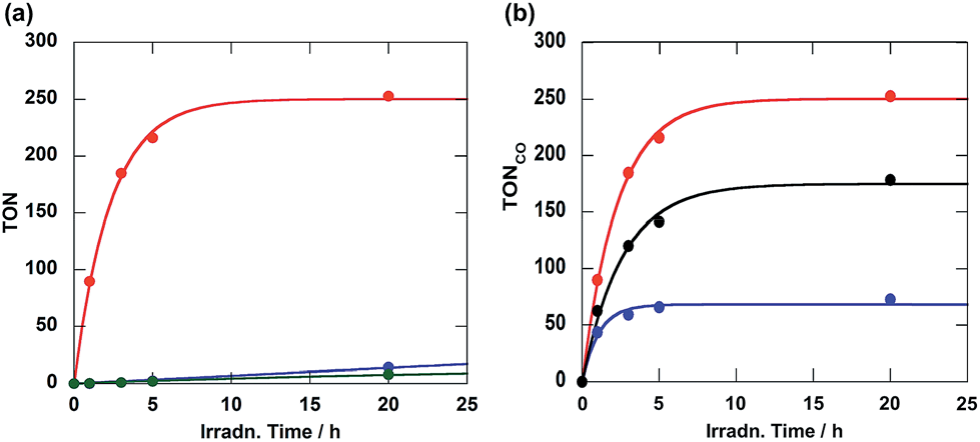
(a) Turnover number of **Ru(CH_2_OCH_2_)Re** for CO (red), HCOOH (green), and H_2_ (blue) formation and (b) those of **Ru(CH_2_OCH_2_)Re** (red), **Ru(CH_2_SCH_2_)Re** (black), and **Ru(CH_2_CH_2_CH_2_)Re** (blue) for CO formation as a function of irradiation time. DMF–TEOA (5 : 1) solutions containing 0.05 mM of the diad and 0.1 M of BNAH were irradiated at *λ* > 500 nm under a CO_2_ atmosphere.

As a typical example of a photocatalytic reaction, a DMF–triethanolamine (TEOA) (5 : 1, v/v) solution containing **Ru(CH_2_OCH_2_)Re** (0.05 mM) and BNAH (0.1 M) as the reductant was irradiated under a CO_2_ atmosphere at wavelengths greater than 500 nm; these wavelengths could be absorbed only by the Ru unit. Carbon monoxide was selectively produced along with very small amounts of H_2_ and HCOOH (Fig. 2a). The turnover number of CO formation (TON_CO_) was as high as 253 for 20 h of irradiation. The quantum yield of CO formation (*Φ*_CO_) was 0.18, which was determined using monochromic light at *λ* = 480 nm (light intensity: 4.3 × 10^–9^ einstein s^–1^). Notably, this *Φ*_CO_ achieved with **Ru(CH_2_OCH_2_)Re** is the highest value among the reported photocatalytic systems in which BNAH was used as the reductant. The other diads also gave CO selectively; the CO formation achieved using each diad is compared in Fig. 2b. The photocatalytic activity of **Ru(CH_2_OCH_2_)Re** (*Φ*_CO_ = 0.18, TON_CO_ = 253) was greater than that of **Ru(CH_2_CH_2_CH_2_)Re** (*Φ*_CO_ = 0.10, TON_CO_ = 178); however, both the efficiency and stability of **Ru(CH_2_SCH_2_)Re** (*Φ*_CO_ = 0.09, TON_CO_ = 73) were lower than those of **Ru(CH_2_CH_2_CH_2_)Re**.

**Fig. 3:**
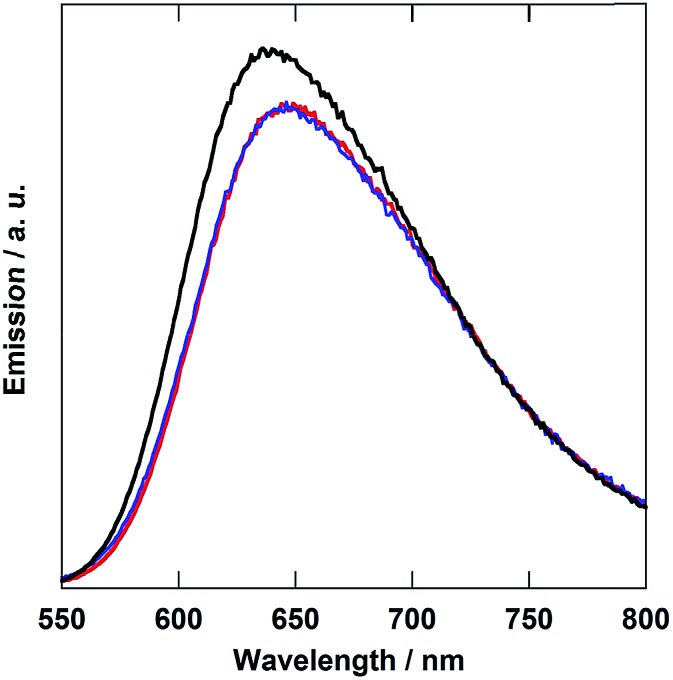
The emission spectra of **Ru(CH_2_OCH_2_)Re** (red), **Ru(CH_2_SCH_2_)Re** (blue), and **Ru(CH_2_CH_2_CH_2_)Re** (black) collected at 25 °C in DMF; the spectra are normalized to the absorbance of the solutions at *λ*_excitation_ = 456 nm.

Fig. 3 shows the emission spectra of the diads. In all cases, broad emission from the ^3^MLCT excited state of the Ru unit was observed. The emission maxima of both **Ru(CH_2_OCH_2_)Re** and **Ru(CH_2_SCH_2_)Re** were red-shifted by 10 nm compared to that of **Ru(CH_2_CH_2_CH_2_)Re**. The emission quantum yields (*Φ*_em_) and emission lifetimes (*τ*_em_) of **Ru(CH_2_OCH_2_)Re** and **Ru(CH_2_SCH_2_)Re** decreased compared to those of **Ru(CH_2_CH_2_CH_2_)Re** according to the energy-gap law (Table 1). These emission properties of the Ru units in both **Ru(CH_2_OCH_2_)Re** and **Ru(CH_2_CH_2_CH_2_)Re** were very similar to those of their model mononuclear complexes, *i.e.*, **Ru(CH_2_OCH_2_)** and **Ru**, respectively (Table 1). These similarities strongly suggest that the difference between the emission properties of **Ru(CH_2_OCH_2_)Re** and **Ru(CH_2_CH_2_CH_2_)Re** was mainly caused by the introduction of the oxygen atom into the bridging ligand; thus, introduction of the Re unit into the opposite side did not strongly affect the emission properties in either case. This result also suggests that intramolecular quenching of the ^3^MLCT of the Ru unit by electron and/or energy transfer to the Re unit did not proceed or proceeded with very low efficiency.

**Table 1:** Emission properties of the metal complexes and quenching rates of the emission by BNAH^*a*^

Complex	*λ*_em_ ^*a*^/nm	*Φ*_em_ ^*a*^	*τ*_em_ ^*a*^/ns	*k*_q_ *τ* ^*b*^ ^,^ ^*c*^/M^–1^ s^–1^	*k*_q_/10^7^ M^–1^	*η*_q_ ^*d*^
**Ru(CH_2_OCH_2_)Re**	649	0.087	735	42.1	5.73	0.82
**Ru(CH_2_SCH_2_)Re**	649	0.087	734	36.3	4.95	0.78
**Ru(CH_2_CH_2_CH_2_)Re**	639	0.097	761	21.0	2.76	0.67
**Ru(CH_2_OCH_2_)Re(CO)_3_(X)**	645	0.119^*b*^	724^*b*^	14.0	1.92	0.58
X = ^–^OC(O)OC_2_H_4_N(C_2_H_4_OH)_2_						
**Ru**	639	0.086	760	—	—	—
**Ru(CH_2_OCH_2_)**	649	0.092	747	—	—	—

^*a*^DMF was used as the solvent, and the excitation wavelength was 456 nm.

^*b*^DMF–TEOA (5 : 1 v/v) was used as the solvent.

^*c*^The excitation wavelength was 520 nm.

^*d*^Quenching fraction with 0.1 M BNAH, *η*_q_ = 1 – 1/(1 + *k*_q_*τ*[BNAH]).

Emission from the Ru units in all of the diads was reductively quenched by BNAH, and the Stern–Volmer plots of the emission quenching exhibited good linearity (Fig. S1, ESI†). Table 1 summarizes the quenching rate constants of the emission from the Ru units (*k*_q_) and the quenching fractions with 0.1 M BNAH (*η*_q_). Under the photocatalytic reaction conditions, the *η*_q_ values of **Ru(CH_2_OCH_2_)Re** and **Ru(CH_2_SCH_2_)Re** were approximately 10% higher than that of **Ru(CH_2_CH_2_CH_2_)Re**. This result strongly suggests that the oxidizing powers of the excited Ru units in **Ru(CH_2_OCH_2_)Re** and **Ru(CH_2_SCH_2_)Re** were improved compared with that of the excited Ru units in **Ru(CH_2_CH_2_CH_2_)Re**, likely because of the high electronegativities of the heteroatoms introduced into the bridging ligands. The increased quenching fraction should be partly responsible for improving the photocatalytic activity of **Ru(CH_2_OCH_2_)Re**. However, the quenching fraction of **Ru(CH_2_OCH_2_)Re** increased only 1.2-fold compared with that of **Ru(CH_2_CH_2_CH_2_)Re**, whereas the quantum yield of CO formation in the case of **Ru(CH_2_OCH_2_)Re** increased by 1.7 times compared with that in the case of **Ru(CH_2_CH_2_CH_2_)Re**. Other factors likely contribute to the improvement of the photocatalytic activity of **Ru(CH_2_OCH_2_)Re**. In addition, both the *Φ*_CO_ and TON_CO_ in the case of **Ru(CH_2_SCH_2_)Re** were lower compared to those in the case of **Ru(CH_2_CH_2_CH_2_)Re**, even though the *η*_q_ of **Ru(CH_2_SCH_2_)Re** was greater than that of **Ru(CH_2_CH_2_CH_2_)Re**.

**Fig. 4:**
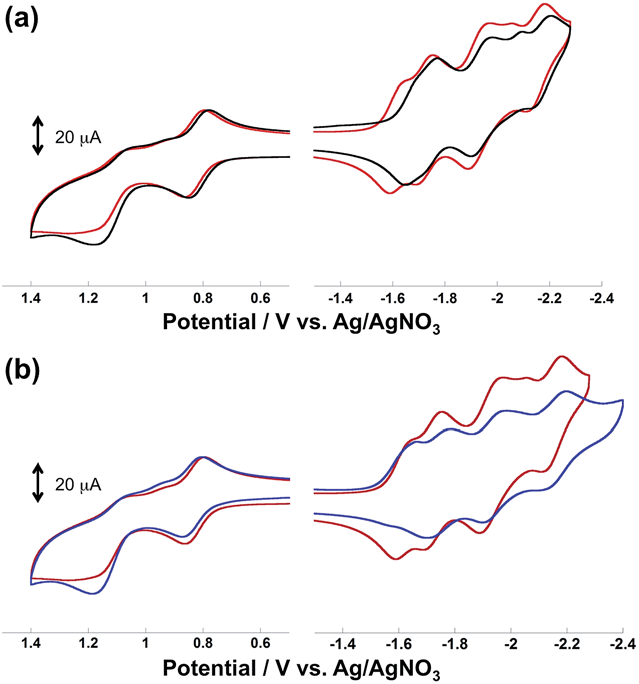
Cyclic voltammograms of (a) **Ru(CH_2_OCH_2_)Re** (red) and **Ru(CH_2_CH_2_CH_2_)Re** (black), and (b) **Ru(CH_2_SCH_2_)Re** (blue) and **Ru(CH_2_OCH_2_)Re** (red) measured in MeCN containing Et_4_NBF_4_ (0.1 M) as the supporting electrolyte.

**Fig. 5:**
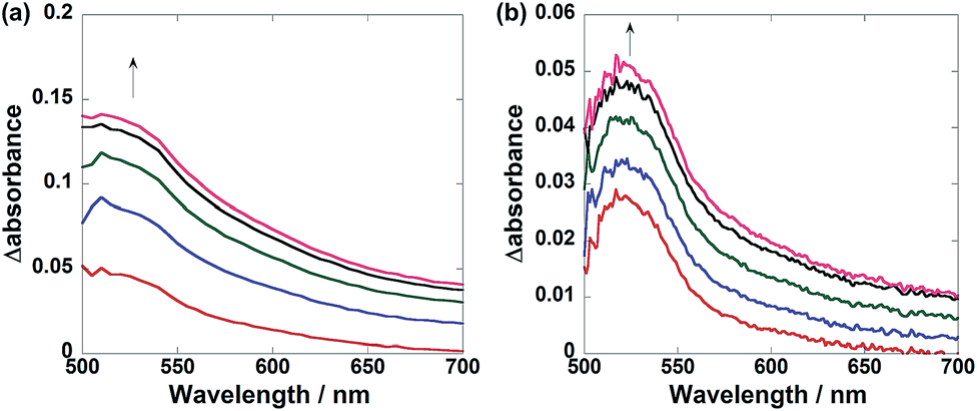
*In situ* differential UV-vis absorption spectra of the reaction solutions before and after irradiation: (a) **Ru(CH_2_OCH_2_)Re**, where the irradiation time was 840–1080 s at intervals of 60 s; (b) **Ru(CH_2_CH_2_CH_2_)Re**, where the irradiation time was 2640–2760 s at intervals of 30 s. DMF–TEOA (5 : 1) solutions containing BNAH (0.1 M) and the complex (0.3 mM) were irradiated under a CO_2_ atmosphere at *λ* = 480 nm with a light intensity of 3.2 × 10^–9^ einsteins^–1^.

Fig. 4 illustrates the cyclic voltammograms (CVs) of the diads measured in MeCN containing Et_4_NBF_4_ (0.1 M) as a supporting electrolyte. In the case of **Ru(CH_2_OCH_2_)Re**, as shown in Fig. 5a, five reduction waves were observed in the cathodic scan; these waves were respectively attributed, from left to right, to the ligand-based reduction of the Re unit, the first ligand-based reduction of the Ru unit, the second ligand-based reduction of the Ru unit, the Re-centered reduction, and the third ligand-based reduction of the Ru unit on the basis of comparison with the corresponding model complexes. All of the reduction waves except for the Re-centered reduction were reversible. In the anodic scan, two oxidation waves were observed, the reversible wave at *E*_1/2_ = 0.82 V and the irreversible wave at *E*_p_ = 1.21 V were attributed to the Ru-centered (Ru(ii)/Ru(iii)) and the Re-centered oxidation (Re(i)/Re(ii)), respectively.

All redox waves in the CV of **Ru(CH_2_CH_2_CH_2_)Re** were similarly observed but negatively shifted compared to those of **Ru(CH_2_OCH_2_)Re** (Fig. 4a). In the case of **Ru(CH_2_SCH_2_)Re**, however, the ligand-based reduction of the Re unit was irreversible and the Re-centered reduction wave could not be identified (Fig. 4b). This result suggests that the one-electron-reduced species (OERs) of **Ru(CH_2_SCH_2_)Re** was less stable than the corresponding OERs of the other diads. The first reduction waves of the Re units in both **Ru(CH_2_OCH_2_)Re** and **Ru(CH_2_CH_2_CH_2_)Re** were observed at potentials similar to those of the corresponding model complexes, *i.e.*, **Re(CH_2_OCH_2_)** and **Re** (Table 2). This observation also indicates that the Ru unit did not exert a strong electronic effect on the Re units through the bridging ligand. Therefore, the positive shifts of the redox potentials of **Ru(CH_2_OCH_2_)Re** compared to those of **Ru(CH_2_CH_2_CH_2_)Re**, as described above, are attributable mainly to the electron-withdrawing ability of the oxygen atom in the bridging ligand, which lowers the HOMO and LUMO energies of the Ru and Re units.

**Table 2:** Electrochemical properties of the metal complexes

Complex	*E*_1/2_ (Δ*E*/mV)/V *vs.* Ag/AgNO_3_						
	Ligand based reduction of the Ru unit			[Re(L/L^–^)]	[Re^0/I^]^*a*^	[Ru^II/III^]	[Re^I/II^]^*a*^
**Ru(CH_2_OCH_2_)Re**	–1.73 (61)	–1.93 (103)	–2.15 (76)	–1.62 (66)	–2.00	+0.82 (70)	+1.21
**Ru(CH_2_SCH_2_)Re**	–1.73 (110)	–1.94 (100)	–2.15 (110)	–1.67^*a*^	—^*b*^	+0.84 (70)	+1.19
**Ru(CH_2_CH_2_CH_2_)Re**	–1.76 (50)	–1.94 (90)	–2.16 (92)	–1.67 (40)	–2.10	+0.81 (70)	+1.15
**Ru(CH_2_OCH_2_)**	–1.72 (73)	–1.91 (72)	–2.15 (70)			+0.84 (76)	
**Ru** ^*c*^	–1.74 (76)	–1.93 (71)	–2.16 (71)			+0.82 (67)	
**Re(CH_2_OCH_2_)**				–1.62 (60)	–2.12		+1.15
**Re** ^*c*^				–1.67 (65)	–2.22		+1.15

^*a*^Peak potential of the irreversible wave.

^*b*^The wave was not identified.

^*c*^From ref. 8*e*.

The first reduction of **Ru(CH_2_OCH_2_)** was observed at a potential 20 mV more positive than that of **Ru**; however, the first reduction of **Re(CH_2_OCH_2_)** was 50 mV more positive than that of **Re** (Table 2). This discrepancy indicates that the introduction of an oxygen atom into the bridging ligand more strongly affected the LUMO energy of the Re unit than that of the Ru unit and that, in the case of **Ru(CH_2_OCH_2_)Re**, the driving force of intramolecular electron transfer from the OERs of the Ru unit to the Re unit was greater than that of **Ru(CH_2_CH_2_CH_2_)Re**.

These results are consistent with the differences between the UV-vis absorption spectra of the solutions during the photocatalytic reactions in the presence of **Ru(CH_2_OCH_2_)Re** and **Ru(CH_2_CH_2_CH_2_)Re** (Fig. 5). In the case of **Ru(CH_2_CH_2_CH_2_)Re**, a relatively sharp absorption band with a maximum at 520 nm appeared during irradiation (Fig. 5a). The corresponding absorption band of **Ru(CH_2_OCH_2_)Re** was broader, with relatively stronger absorption at wavelengths longer than ∼560 nm (Fig. 5b). Both spectra were fitted by a linear combination of the absorption spectra of the OERs of **Ru** and **Re**, which were obtained by the flow electrolysis method (Fig. S2, ESI†). Using these data, we calculated the ratios of the OERs between the Ru and Re units to be 1 : 1.3 in the case of **Ru(CH_2_CH_2_CH_2_)Re**, and 1 : 9.3 in the case of **Ru(CH_2_OCH_2_)Re** (Fig. S3, ESI†). These results strongly indicate that the added electron was more localized on the Re unit in the OERs of **Ru(CH_2_OCH_2_)Re** than that of **Ru(CH_2_CH_2_CH_2_)Re** because of the greater driving force of the intramolecular electron transfer from the Ru unit to the Re unit. This greater driving force should be another reason for the higher photocatalytic efficiency of **Ru(CH_2_OCH_2_)Re**.

**Fig. 6:**
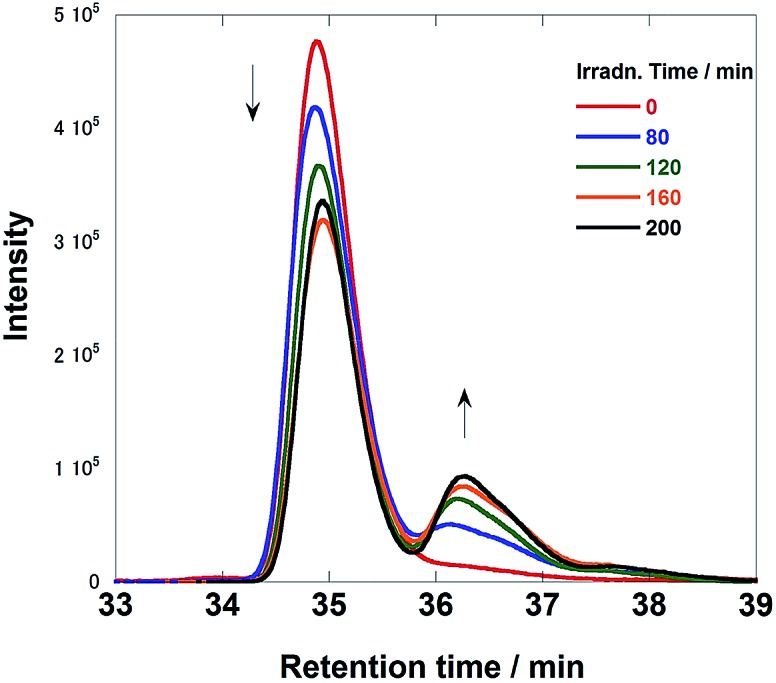
Size exclusion chromatograms of the solutions before and after the photocatalytic reaction using **Ru(CH_2_OCH_2_)Re** (irradiation times: 0, 80, 120, 160, and 200 min). The eluent was MeCN–MeOH (1 : 1 v/v) containing 0.5 M CH_3_COONH_4_; two Shodex PROTEIN KW402.5 columns were used with a KW-LG guard column under the following conditions: flow rate, 0.2 mL min^–1^; column temperature, 40 °C; detection wavelength, 390 nm. The reaction conditions were the same as those described in Fig. 5.

Fig. 6 illustrates size-exclusion chromatograms of the reaction solution in the case of **Ru(CH_2_OCH_2_)Re**. Before irradiation, only a single peak attributed to **Ru(CH_2_OCH_2_)Re** was observed at 34.9 min. The irradiation induced a decrease in the intensity of the peak of **Ru(CH_2_OCH_2_)Re** with the appearance of a new peak at 36.1 min; after 120 min of irradiation, a photostationary state, where the ratio of the peak areas was approximately 3 : 1, was observed. The electrospray ionization (ESI) mass spectra of the eluents collected at 36.1 min showed a peak attributable to [(dmb)_2_Ru(CH_2_OCH_2_)Re(CO)_3_(CH_3_COO^–^)]^2+^, whose CH_3_COO^–^ group likely originated from the eluent (MeCN–MeOH (1 : 1 v/v) containing 0.05 M CH_3_COONH_4_) at *m*/*z* = 591 (Fig. S4, ESI†). This result strongly suggests that the Re unit in some **Ru(CH_2_OCH_2_)Re** complexes was converted into a tricarbonyl complex, *i.e.*, *fac*-[Re(N⁁N)(CO)_3_(L)] (N⁁N = diimine ligand, L = monodentate ligand), during the first stage of the photocatalytic reaction.

**Fig. 7:**
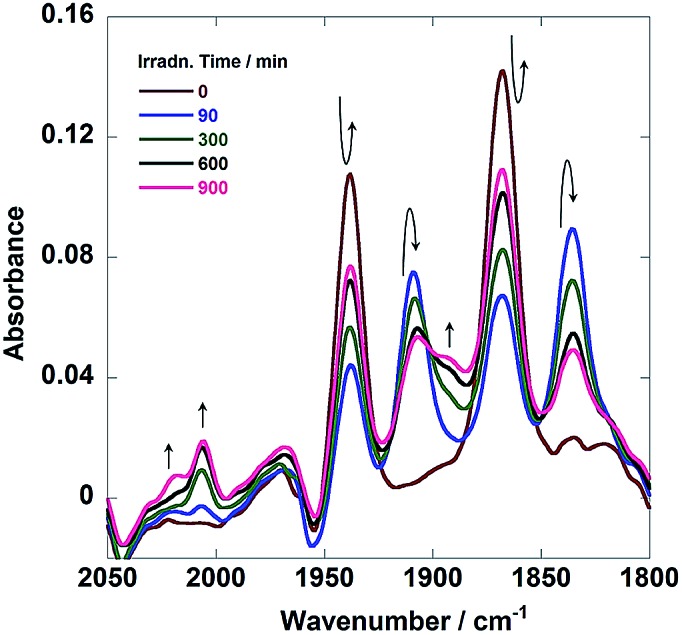
*In situ* IR spectra of a DMF–TEOA (5 : 1 v/v) solution containing **Ru(CH_2_OCH_2_)Re** (1 mM) and BNAH (0.1 M) during photoirradiation at *λ* = 480 nm under a CO_2_ atmosphere.

Fig. 7 shows *in situ* IR spectral changes of a solution during the photocatalytic reaction. The concentration of the diad was greater than that used in the case of Fig. 6 because of the lower sensitivity of the IR spectrometer compared to that of the size exclusion chromatograph. Immediately after the irradiation was initiated, the CO-stretching bands corresponding to the Re unit of **Ru(CH_2_OCH_2_)Re** (*ν*_CO_ = 1938 and 1868 cm^–1^) decreased in intensity, whereas a pair of bands of the corresponding OERs (*ν*_CO_ = 1909 and 1836 cm^–1^) increased in intensity.^10^ Further irradiation resulted in the formation of new bands attributable to two kinds of diads with a Re tricarbonyl unit (*ν*_CO_ = 2018, 2006, and ∼1890 cm^–1^; other *ν*_CO_ bands associated with these species should be observed, but they were overlapped by the bands of other species). These species were identified as diads with two types of the Re units: *fac*-[Re(N⁁N)(CO)_3_{OC_2_H_4_N(C_2_H_4_OH)_2_}] with a deprotonated TEOA ligand, and its CO_2_-inserted product, *fac*-[Re(N⁁N)(CO)_3_{OC(O)OC_2_H_4_N(C_2_H_4_OH)_2_}], where both TEOA and CO_2_ molecules are captured to form a carbonate ester ligand. These structural assignments are based on the similarities of these species to the corresponding Re mononuclear complexes, *i.e.*, *fac*-[Re(bpy)(CO)_3_{OC_2_H_4_N(C_2_H_4_OH)_2_}] (*ν*_CO_ = 2006, 1897, 1881 cm^–1^) and *fac*-[Re(bpy)(CO)_3_{OC(O)OC_2_H_4_N(C_2_H_4_OH)_2_}] (*ν*_CO_ = 2020, 1915, 1892 cm^–1^) (bpy = 2,2′-bipyridine), reported in our previous paper.^11^ As described below, the IR spectra of authentic samples of the diads with either a ^–^OC_2_H_4_N(C_2_H_4_OH)_2_ or a ^–^OC(O)OC_2_H_4_N(C_2_H_4_OH)_2_ ligand, *i.e.*, **Ru(CH_2_OCH_2_)Re(CO)_3_(X)** (X = ^–^OC_2_H_4_N(C_2_H_4_OH)_2_ or ^–^OC(O)OC_2_H_4_N(C_2_H_4_OH)_2_) also showed similar CO-stretching bands (Fig. S5, ESI†). These results strongly indicate that **Ru(CH_2_OCH_2_)Re** was partially converted into **Ru(CH_2_OCH_2_)Re(CO)_3_(X)** in the first stage of the photocatalytic reaction (eqn (1)).1
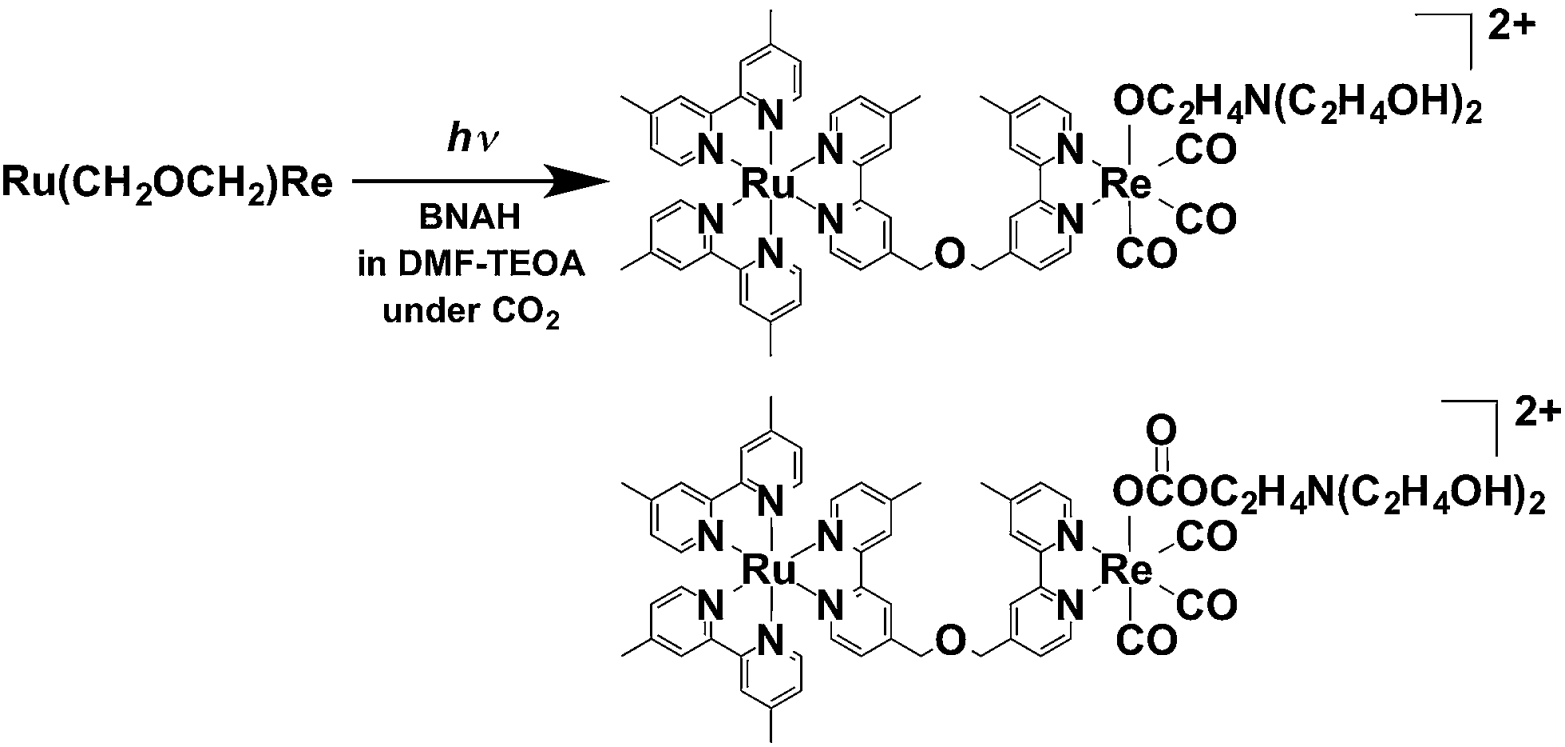



**Fig. 8:**
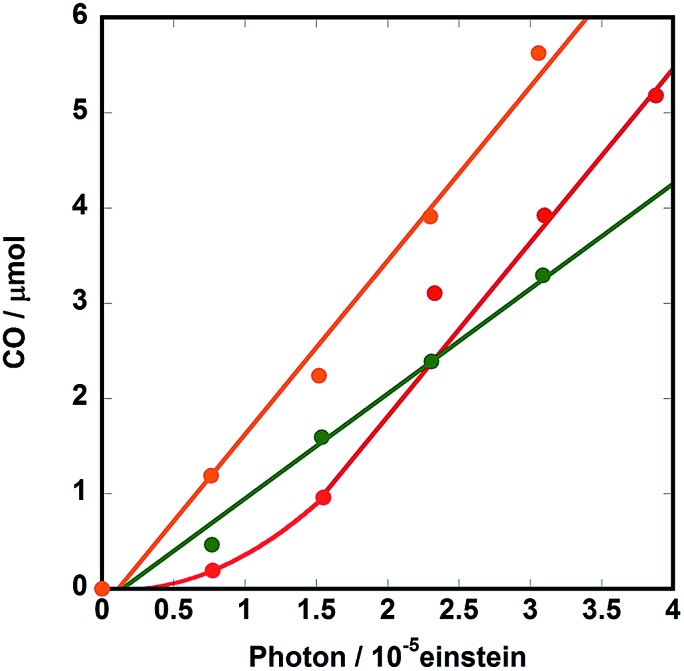
Photocatalytic CO formation using DMF-TEOA (5 : 1 v/v) solutions containing 0.1 M of BNAH and 0.3 mM of **Ru(CH_2_OCH_2_)Re** (red), **Ru(CH_2_OCH_2_)Re(CO)_3_(X)** (green), or a 3 : 1 mixture of **Ru(CH_2_OCH_2_)Re** and **Ru(CH_2_OCH_2_)Re(CO)_3_(X)** (orange). The reaction conditions were same as those described in Fig. 5.

Further irradiation resulted in a photostationary state, where the reaction solution contained both **Ru(CH_2_OCH_2_)Re** and **Ru(CH_2_OCH_2_)Re(CO)_3_(X)** in an approximately 3 : 1 ratio, as described above. Fig. 8 illustrates the initial stage of photocatalytic CO formation at lower light intensity (3.2 × 10^–9^ einstein s^–1^) than that used in the case of Fig. 2. An induction period of CO formation was observed in the case of **Ru(CH_2_OCH_2_)Re**, which strongly indicates that **Ru(CH_2_OCH_2_)Re(CO)_3_(X)** played an important role in the photocatalytic reaction. Therefore, we synthesized **Ru(CH_2_OCH_2_)Re(CO)_3_(X)** by reacting **Ru(CH_2_OCH_2_)Re(CO)_3_(MeCN)**, TEOA, and CO_2_^11^ and investigated the photocatalytic ability of **Ru(CH_2_OCH_2_)Re(CO)_3_(X)**; the results are also shown in Fig. 8 (green). The clear induction period of CO formation disappeared; however, the quantum yield of CO formation (*Φ*_CO_ = 0.12) was lower than that achieved with **Ru(CH_2_OCH_2_)Re** (*Φ*_CO_ = 0.18, red in Fig. 8). Given these results, we examined the photocatalytic ability of a 3 : 1 mixed system of **Ru(CH_2_OCH_2_)Re** and **Ru(CH_2_OCH_2_)Re(CO)_3_(X)**, which is a similar ratio observed in the photostationary state when only **Ru(CH_2_OCH_2_)Re** was used as the photocatalyst (Fig. 6). As shown in Fig. 8 (orange), no clear induction period was observed and the quantum yield of CO formation achieved using this system (*Φ*_CO_ = 0.19) was similar to that achieved using **Ru(CH_2_OCH_2_)Re** after the induction period (*Φ*_CO_ = 0.18). Therefore, we conclude that **Ru(CH_2_OCH_2_)Re** and **Ru(CH_2_OCH_2_)Re(CO)_3_(X)** played important but different roles in the photocatalytic reaction.

**Fig. 9:**
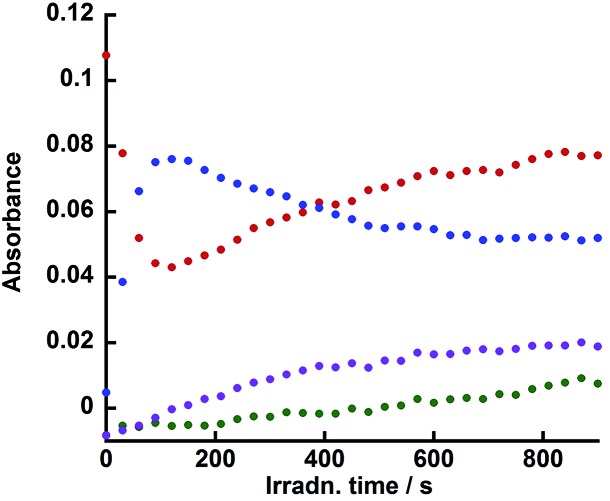
Changes in the intensities of the absorbance peaks in the *in situ* IR spectra (Fig. 7): **Ru(CH_2_OCH_2_)Re(CO)_3_(X)** (green, 2018 cm^–1^, X = ^–^OC(O)OC_2_H_4_N(C_2_H_4_OH)_2_; purple, 2006 cm^–1^, X = ^–^OC_2_H_4_N(C_2_H_4_OH)_2_), **Ru(CH_2_OCH_2_)Re** (red, 1938 cm^–1^), and the OER species of **Ru(CH_2_OCH_2_)Re** (blue, 1909 cm^–1^). See Fig. 7.

Fig. 9 illustrates the rise and decay curves of the FT-IR absorption peaks (Fig. 7) attributed to each diad in the reaction solution during irradiation. However, immediately after the irradiation was started, the OERs of the Re unit in **Ru(CH_2_OCH_2_)Re** (blue) formed rapidly and its concentration decreased between 120 s and 600 s of irradiation time, the non-reduced **Ru(CH_2_OCH_2_)Re** (red) was recovered. Further irradiation did not induce a change in the ratio between the OERs and non-reduced species. The decrease of the concentration of the OERs appeared to occur after **Ru(CH_2_OCH_2_)Re(CO)_3_(X)** (green and purple) was accumulated in the reaction solution. These results indicate that efficient electron transfer from the OERs of **Ru(CH_2_OCH_2_)Re** to **Ru(CH_2_OCH_2_)Re(CO)_3_(X)** and/or an intermediate produced from the OERs of **Ru(CH_2_OCH_2_)Re(CO)_3_(X)** occurred during the steady-state CO formation in the photocatalytic reaction.

**Scheme 1:**
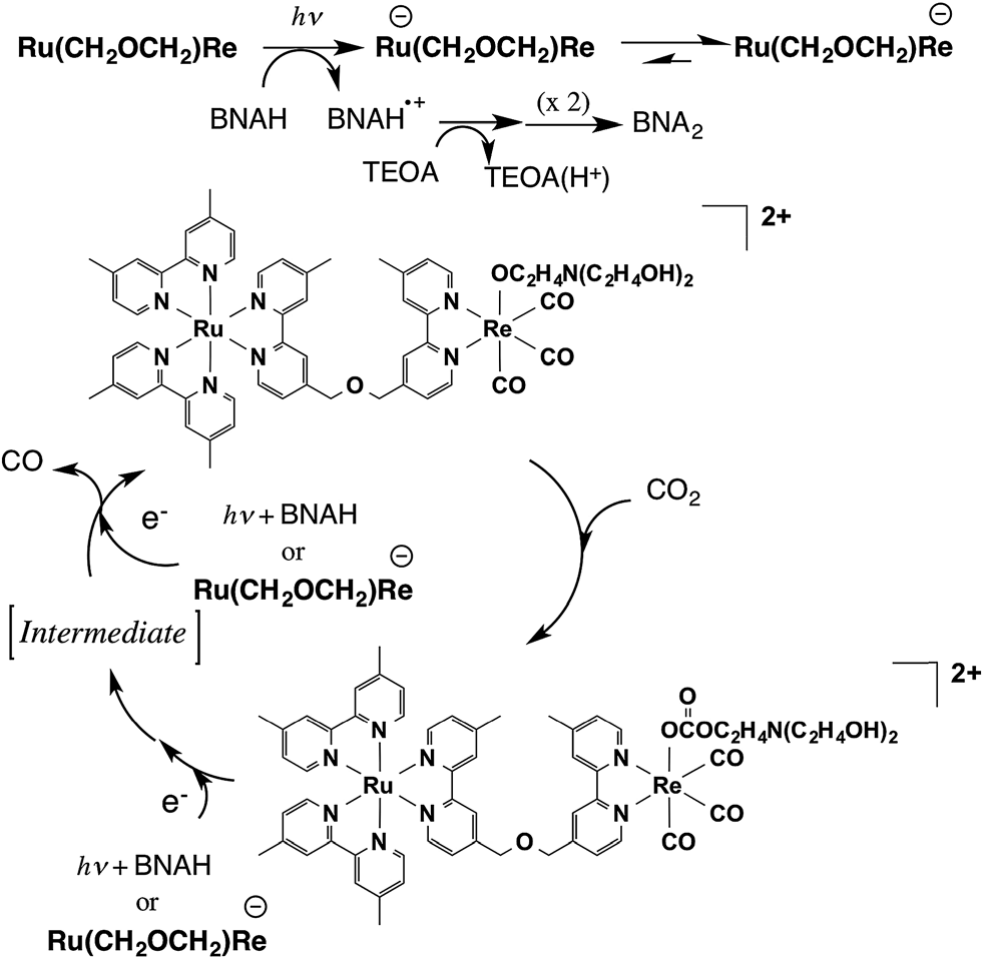
Partial reaction mechanism of photocatalytic CO_2_ reduction by **Ru(CH_2_OCH_2_)Re**.

Scheme 1 summarizes the possible mechanism for the photocatalytic reaction involving **Ru(CH_2_OCH_2_)Re**. The Ru unit is selectively excited, and the resulting ^3^MLCT excited state is reductively quenched by BNAH to give the OER species of the Ru unit of **Ru(CH_2_OCH_2_)Re**. Rapid intramolecular electron transfer proceeds from the OERs of the Ru unit to the Re unit to give the OER species of the Re unit of **Ru(CH_2_OCH_2_)Re**. Rapid abstraction of a proton from the one-electron-oxidized BNAH by TEOA and dimerization have been reported to proceed to give BNA_2_. In the first stage of the photocatalytic reaction, approximately a quarter of the added **Ru(CH_2_OCH_2_)Re** was efficiently converted into **Ru(CH_2_OCH_2_)Re(CO)_3_(X)** (X = ^–^OC_2_H_4_N(C_2_H_4_OH)_2_ or ^–^OC(O)OC_2_H_4_N(C_2_H_4_OH)_2_), which was the real photocatalyst for CO_2_ reduction. **Ru(CH_2_OCH_2_)Re**, which remained in the solution, functioned as a redox photosensitizer that supplied electrons to **Ru(CH_2_OCH_2_)Re(CO)_3_(X)** and/or the intermediate during the photocatalytic reaction. Notably, the Ru unit of **Ru(CH_2_OCH_2_)Re(CO)_3_(X)** should serve as another redox photosensitizer (Table 1, Fig. S6, ESI†) because **Ru(CH_2_OCH_2_)Re(CO)_3_(X)** alone can function as a relatively efficient photocatalyst for CO_2_ reduction, though its *Φ*_CO_ is lower than that of the mixed system. Because the absorption band attributable to the OER species of **Ru(CH_2_OCH_2_)Re(CO)_3_(X)** was not detected in the UV-vis absorption spectrum of the reaction solution during the photocatalytic reaction, the photochemically accepted electron in the Ru unit should rapidly transfer to the Re unit and then participate in the reduction of CO_2_. The electrons for the second reduction process, where an electron was accepted by the Re unit of the intermediate forming from the OER species of **Ru(CH_2_OCH_2_)Re(CO)_3_(X)** to produce CO, originated from two possible sources: the reduced Ru unit in the intermediate or the reduced **Ru(CH_2_OCH_2_)Re**. The previously described results indicate that the reduction efficiency with the latter species was better than that with the former species; *i.e.*, the reduction of the intermediate should be a rate-limiting process in the photocatalytic reduction of CO_2_*via***Ru(CH_2_OCH_2_)Re(CO)_3_(X)** under the investigated reaction conditions. Because similar photocatalytic behavior of **Ru(CH_2_CH_2_CH_2_)Re** was observed (Fig. S7–S9, ESI†), the photocatalytic reaction involving **Ru(CH_2_CH_2_CH_2_)Re** should proceed through a mechanism similar to that for **Ru(CH_2_OCH_2_)Re**.

**Fig. 10:**
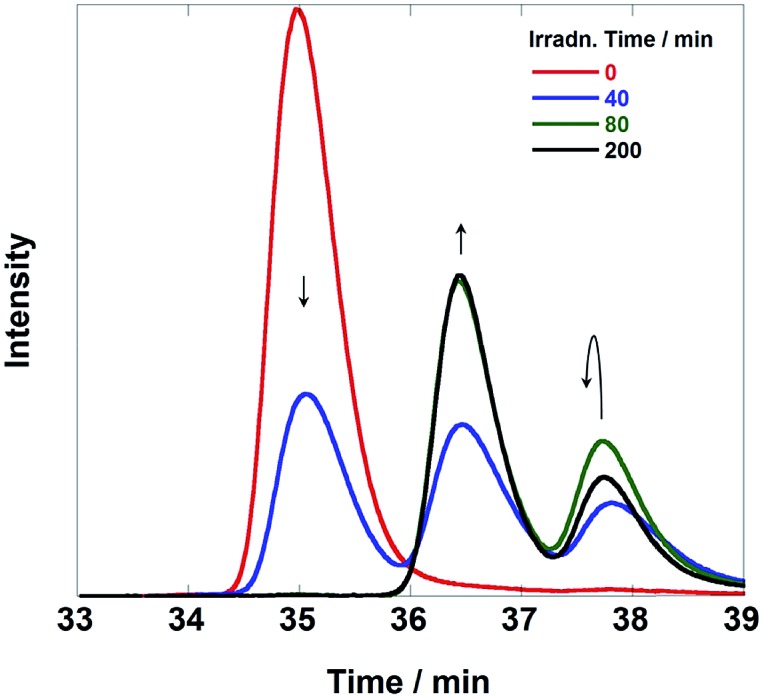
Size exclusion chromatograms of the solutions before and after the photocatalytic reaction using **Ru(CH_2_SCH_2_)Re** (irradiation times: 0, 40, 80, and 200 min). The reaction and analytical conditions were the same as those described in Fig. 6.

As previously described, the durability of **Ru(CH_2_SCH_2_)Re** was much lower than that observed for the other diads. Fig. 10 shows SEC analysis data of the reaction solution; these chromatograms clearly indicate that **Ru(CH_2_SCH_2_)Re** completely disappeared after irradiation for 80 min and that two new species with smaller sizes formed. The LC-MS analysis data (Fig. S10, ESI†) indicated that the species detected at retention times of 36.5 min and 37.8 min were Ru(ii) mononuclear complexes, *i.e.*, **Ru(CH_2_OCH_2_)**, **Ru**, and [Ru(dmb)_2_(**CS^–^**)]^+^ (CS^–^ = MebpyCH_2_S^–^), and Re(i) mononuclear complexes, *i.e.*, **Re**, [Re(**CH_2_OCH_2_**)(CO)_2_{P(*p*-FPh_3_)_2_}_2_]^+^, and [Re(dmb) (CO)_2_{P(*p*-FPh)_3_}(MeCN)]^+^ (MeCN was contained in the eluent), respectively. Therefore, **Ru(CH_2_SCH_2_)Re** was completely cleaved to give the mononuclear complexes in the first stage of the reaction, which should lower both the efficiency and durability of the photocatalysis of **Ru(CH_2_SCH_2_)Re**.

## Experimental

### General procedures

UV-vis absorption spectra were recorded on a JASCO V-565 spectrophotometer. ^1^H NMR spectra were recorded on a JEOL AL300 (300 MHz) spectrometer. The residual proton of the deuterated solvent (acetone-*d*_6_ or acetonitrile-*d*_3_) was used as an internal standard. Emission spectra were measured at 25 ± 0.1 °C under an Ar atmosphere using a JASCO FP-6500 spectrofluorometer. Emission quantum yields were obtained with a Hamamatsu photonics C-9920-02 integrating sphere and a multi-photodiode-array detector. Emission lifetimes were measured with a Horiba FluoroCube time-correlated single-photon counting system (the excitation source was an LED pulse lamp (456 nm) with an instrumental response time of less than 0.1 ns). In the quenching experiments, the changes in the emission intensities of solutions containing a complex were monitored in the presence of various amounts of the quencher, BNAH. Quenching rate constants (*k*_q_) were calculated from linear Stern–Volmer plots. Electrospray ionization-mass spectroscopy (ESI-MS) was recorded on a Shimadzu LCMS-2010A mass spectrometer using MeCN or MeOH as the mobile phase. The redox potentials of the complexes in a MeCN solution were measured using a cyclic voltammetric technique. The CVs were recorded on an ALS/CHI CHI-620 electrochemical analyzer using Et_4_NBF_4_ (0.1 M) as the supporting electrolyte, a glassy-carbon disk working electrode (3 mm diameter), an Ag/AgNO_3_ (0.01 M) reference electrode, and a Pt counter electrode. The scan rate was 200 mV s^–1^. Analytical SEC^12^ was conducted using an HPLC system consisting of a JASCO 880-PU pump, a pair of Shodex PROTEIN KW402.5 columns, a KW-LG guard column, a Rheodyne 7125 injector, and a JASCO MD-2010 plus UV-Vis photodiode-array detector. The column temperature was maintained at 40 °C using a JASCO 860-CO column oven. A mixed solution of MeCN–MeOH (1 : 1 v/v) containing CH_3_COONH_4_ (0.5 mM) was used as the mobile phase. In the case of HPLC(SEC)-MS analyses, a HPLC system consisting of the SEC columns and the guard column, a Rheodyne 7125 injector, a Shimadzu FCV-10ALVP gradient unit, and a Shimadzu LC-10ADVP pump was connected before the ESI-MS detector. The column temperature was maintained at 40 °C using a Shimadzu SPD CTO-10ACVP oven. A MeCN–MeOH (1 : 1 v/v) mixed solution containing CH_3_COONH_4_ (0.05 mM) was used as the mobile phase. Isolation of the diads was achieved by preparative SEC using a pair of Shodex PROTEIN KW 2002.5 columns, a KW-LG guard column, and a JAI LC-9201 recycling preparative HPLC apparatus with a JASCO 870-UV detector. A MeCN–MeOH (1 : 1 v/v) mixed solution containing CH_3_COONH_4_ (0.15 mM) was used as the mobile phase.

### Photocatalytic reactions

Photocatalytic reactions were performed on a 4 mL DMF–TEOA (5 : 1 v/v) mixed solution containing the diad (0.05 mM) and BNAH (0.1 M) in an 11 mL test tube (i.d. 8 mm). After the solution was purged with CO_2_ for 15 min, it was irradiated in a merry-go-round irradiation apparatus using *λ* > 500 nm light from a high-pressure Hg lamp equipped with a uranyl glass and a K_2_CrO_4_ (30% w/w, light pass length: 1 cm) solution filter. The tube was cooled with tap water during irradiation. In the case of quantum yield measurements, the mixed solution containing a higher concentration of the diad (0.3 mM) in a quartz cubic cell (the light pass length: 1 cm) was irradiated using a 480 nm monochromic light from an Ushio UXL-500D-O xenon short-arc lamp equipped with a 480 nm (FWHM: 10 nm) band-pass filter (Asahi Spectra Co.) and a CuSO_4_ solution filter (250 g L^–1^, light pass length: 5 cm). The absorbance of the reaction solution (>3 at 480 nm) during the measurement indicated that most of the irradiated light was absorbed by the diad in the solution. The temperature of the solutions was controlled during irradiation at 25 ± 0.1 °C using an IWAKI CTS-134A constant-temperature system. The incident light intensity was determined using a K_3_Fe(C_2_O_4_)_3_ actinometer.^13^ The gaseous products, *i.e.*, CO and H_2_, were analyzed by GC-TCD (GL Science GC323), and the liquid-phase product, *i.e.*, HCOOH, was analyzed by a capillary electrophoresis system (Otsuka Electronics Co. Capi-33001).

### Materials

Acetonitrile was distilled over P_2_O_5_ three times and then distilled over CaH_2_ immediately before use. DMF was dried over molecular sieves 4A and then distilled under reduced pressure (10–20 mmHg). TEOA was distilled under reduced pressure (<1 mmHg). DMF and TEOA were stored under an Ar atmosphere prior to use. Et_4_NBF_4_ was dried under vacuum at 100 °C for a day prior to use. All other reagents were reagent-grade quality and were used without further purification. BNAH,^14^ the bridging ligands (4′-methyl-[2,2′-bipyridine]-4-yl)-CH_2_XCH_2_-(4′-methyl-[2,2′-bipyridine]-4-yl): X = O (**CH_2_OCH_2_**);^15^ S (**CH_2_SCH_2_**);^15^ CH_2_ (**CH_2_CH_2_CH_2_**),^16^ Ru(dmb)_2_Cl_2_,^17^ and *mer*-[Re(CO)_3_{P(*p*-FPh_3_)}_2_Br]^18^ were synthesized according to the methods described in the literature.

### Synthetic procedures

#### Synthesis of [Re(CO)_2_{P(*p*-FPh)_3_}_2_(CH_2_OCH_2_)](PF_6_)·3H_2_O (**Re(CH_2_OCH_2_)**)

A THF solution (17 mL) containing *mer*-[Re(CO)_3_{P(*p*-FPh_3_)}_2_Br] (197 mg, 0.200 mmol) and AgOTf (54.0 mg, 0.210 mmol) was refluxed for 3 h under an N_2_ atmosphere in the absence of light. After the mixture had cooled to room temperature, the white precipitate was removed by filtration through Celite. After evaporation of the solvent, **CH_2_OCH_2_** (229 mg, 0.599 mmol) was added to the residue and the mixture was dissolved in EtOH (60 mL). The solution was refluxed for 5 h under an N_2_ atmosphere in the absence of light. After evaporation of the solvent, the by-products were removed by column chromatography on an SP Sephadex C-25 ion-exchange column using MeCN–H_2_O (1 : 1 v/v) containing NH_4_PF_6_ as the eluent and then by preparative SEC. Purification of the product was achieved by recrystallization using a MeOH–H_2_O mixed solvent. The yield was 32% (91 mg, 0.065 mmol). Elemental analysis calcd (%) for C_62_H_52_F_12_N_4_O_6_P_3_Re: C. 51.14; H. 3.60; N. 3.85, found: C. 51.18; H. 3.50; N. 3.89. ^1^H-NMR (300 MHz, acetone-*d*_6_) *δ*/ppm: 8.68 (d, *J* = 4.8 Hz, 1H, β-bpy-6), 8.51 (s, 1H, γ-bpy-3), 8.49 (d, *J* = 4.4 Hz, 1H, *α*-bpy-6), 8.41 (s, 1H, β-bpy-3), 8.35 (s, 1H, δ-bpy-3), 8.28 (s, 1H, α-bpy-3), 8.19 (d, *J* = 4.8 Hz, 1H, γ-bpy-6), 8.07 (d, *J* = 3.6 Hz, 1H, δ-bpy-6), 7.44 (d, *J* = 4.8 Hz, 1H, β-bpy-5), 7.38–7.32 (m, 12H, Ph-*o*), 7.28–7.23 (m, 2H, α-bpy-5, γ-bpy-5), 7.12 (t, *J*_HH_ = 8.4 Hz, *J*_FH_ = 8.4 Hz, 12H, Ph-*m*), 7.07 (d, *J* = 5.6 Hz, 1H, δ-bpy-5), 4.84 (s, 2H, β-C*H*_2_), 4.82 (s, 2H, γ-C*H*_2_), 2.46 (s, 6H, α-bpy-C*H*_3_, δ-bpy-C*H*_3_). FT-IR (in CH_2_Cl_2_) *ν*_CO_/cm^–1^: 1868, 1939. ESI-MS (eluent: MeCN) *m*/*z*: 629 ([M – PF_6_^–^ + H^+^]^2+^), 944 ([M – P(*p*-FPh)_3_ – PF_6_^–^]^+^).

#### Synthesis of [(dmb)_2_Ru(CH_2_OCH_2_)](PF_6_)_2_·H_2_O (**Ru(CH_2_OCH_2_)**)

An EtOH–H_2_O (40 mL and 10 mL) mixed solution containing Ru(dmb)_2_Cl_2_ (91.5 mg, 0.159 mmol) and **CH_2_OCH_2_** (184 mg, 0.482 mmol) was refluxed for 8 h under an N_2_ atmosphere in the absence of light. After evaporation of the solvent, water was added to the residue, giving a white precipitate of **CH_2_OCH_2_**; this precipitate was removed by filtration. NH_4_PF_6_ was added to the filtrate to give precipitates, which were recovered by filtration and washed with H_2_O. The product was isolated by column chromatography on an SP Sephadex C-25 ion-exchange column using MeCN–H_2_O (1 : 1 v/v) containing NH_4_PF_6_ as the eluent; the product was subsequently recrystallized from a CH_2_Cl_2_–ether mixed solvent. The yield was 88% (160 mg, 0.14 mmol). Elemental analysis calcd (%) for C_48_H_48_F_12_N_8_O_2_P_2_Ru: C. 49.70; H. 4.17; N. 9.66, found: C. 49.66; H. 4.13; N. 9.59. ^1^H-NMR (300 MHz, acetone-*d*_6_) *δ*/ppm: 8.78 (s, 1H, β-bpy-3), 8.66 (s, 5H, dmb-3, α-bpy-3), 8.62 (d, *J* = 4.5 Hz, 1H, γ-bpy-6), 8.48–8.46 (m, 2H, δ-bpy-6, γ-bpy-3), 8.31 (s, 1H, δ-bpy-3), 7.98 (d, *J* = 5.7 Hz, 1H, β-bpy-6), 7.85–7.81 (m, 5H, α-bpy-6, dmb-6), 7.57 (d, *J* = 4.2 Hz, 1H, β-bpy-5), 7.42–7.35 (m, 6H, γ-bpy-5, dmb-5, α-bpy-5), 7.25 (d, *J* = 4.8 Hz, 1H, δ-bpy-5), 4.92 (s, 2H, β-C*H*_2_), 4.85 (s, 2H, γ-C*H*_2_), 2.55–2.43 (m, 18H, dmb-C*H*_3_, α-bpy-C*H*_3_, δ-bpy-C*H*_3_). ESI-MS (eluent: MeCN) *m*/*z*: 426 ([M – 2PF_6_^–^]^2+^).

The other mononuclear complexes (**Ru(CH_2_SCH_2_)** and **Ru(CH_2_CH_2_CH_2_)**) were synthesized in a manner similar to that used for **Ru(CH_2_OCH_2_)** but with **CH_2_SCH_2_** or **CH_2_CH_2_CH_2_** used instead of **CH_2_OCH_2_** as the bridging ligand.

#### [(dmb)_2_Ru(CH_2_SCH_2_)](PF_6_)_2_ (**Ru(CH_2_SCH_2_)**)

Yield 44%. ^1^H-NMR (300 MHz, acetone-*d*_6_) *δ*/ppm: 8.60 (s, 4H, dmb-3), 8.47 (s, 1H, β-bpy-3), 8.36 (d, *J* = 5.2 Hz, 1H, γ-bpy-6), 8.34 (s, 1H, γ-bpy-3), 8.29 (d, *J* = 4.9 Hz, 1H, δ-bpy-6), 8.26 (s, 1H, α-bpy-3), 8.11 (s, 1H, δ-bpy-3), 7.81 (d, *J* = 5.9 Hz, 1H, β-bpy-6), 7.76–7.71 (m, 5H, dmb-6, α-bpy-6), 7.46 (d, *J* = 5.9 Hz, 1H, β-bpy-5), 7.41–7.25 (m, 6H, α-bpy-5, dmb-5, γ-bpy-5), 7.17 (d, *J* = 4.2 Hz, 1H, δ-bpy-5), 3.94–3.78 (m, 4H, β-C*H*_2_, γ-C*H*_2_), 2.53–2.33 (m, 18H, dmb-C*H*_3_, α-bpy-C*H*_3_, δ-bpy-C*H*_3_). ESI-MS (eluent: MeCN) *m*/*z*: 434 ([M – 2PF_6_^–^]^2+^).

#### [(dmb)_2_Ru(CH_2_CH_2_CH_2_)](PF_6_)_2_ (**Ru(CH_2_CH_2_CH_2_)**)

Yield 34%. ^1^H-NMR (300 MHz, acetone-*d*_6_) *δ*/ppm: 8.67 (s, 1H, β-bpy-3), 8.64 (s, 4H, dmb-3), 8.62 (s, 1H, α-bpy-3), 8.52 (d, *J* = 5.0 Hz, 1H, γ-bpy-6), 8.47 (d, *J* = 5.0 Hz, 1H, δ-bpy-6), 8.29 (s, 2H, γ-bpy-3, δ-bpy-3), 7.86–7.80 (m, 6H, α-bpy-6, β-bpy-6, dmb-6), 7.45 (d, *J* = 5.5 Hz, 1H, β-bpy-5), 7.38–7.36 (m, 5H, α-bpy-5, dmb-5), 7.29–7.25 (m, 2H, γ-bpy-5, δ-bpy-5), 3.00–2.81 (m, 4H, β-C*H*_2_, γ-C*H*_2_), 2.55–2.43 (m, 18H, dmb-C*H*_3_, α-bpy-C*H*_3_, δ-bpy-C*H*_3_), 2.16 (tt, 4H, *J* = 4.6 Hz, 4.6 Hz, –CH_2_–C*H*_2_–CH_2_–). ESI-MS (eluent: MeCN) *m*/*z*: 426 ([M – 3PF_6_^–^]^3+^).

#### [(dmb)_2_Ru(CH_2_OCH_2_)Re{P(*p*-FPh)_3_}_2_](PF_6_)_3_ (**Ru(CH_2_OCH_2_)Re**)

A THF solution (30 ml) containing *mer*-[Re(CO)_3_{P(*p*-FPh_3_)}_2_Br] (86.5 mg, 0.0880 mmol) and AgOTf (23.7 mg, 0.0924 mmol) was refluxed for 4 h under an N_2_ atmosphere in the absence of light. After the mixture had cooled to room temperature, white precipitates were removed by filtration through Celite. After evaporation of the solvent, **Ru(CH_2_OCH_2_)** (51.4 mg, 0.0450 mmol) was added to the residue, and the mixture was dissolved in EtOH (60 mL). The solution was refluxed for 15 h under an N_2_ atmosphere in the absence of light. After evaporation of the solvent, the product was purified by column chromatography on a CM Sephadex C-25 ion-exchange column using MeCN–H_2_O (1 : 1 v/v) containing NH_4_PF_6_ as the eluent; the product was subsequently recrystallized with CH_2_Cl_2_–ether mixed solvent. The yield was 36% (35 mg, 0.016 mmol). Elemental analysis calcd (%) for C_86_H_70_F_24_N_8_O_3_P_5_ReRu: C. 47.78; H. 3.26; N. 5.18. Found: C. 47.52; H. 3.36; N. 5.16. ^1^H-NMR (300 MHz, acetone-*d*_6_) *δ*/ppm: 8.72 (s, 1H, β-bpy-3), 8.62 (s, 5H, α-bpy-3, dmb-3), 8.29 (s, 1H, γ-bpy-3), 8.20 (s, 1H, δ-bpy-3), 8.05 (d, *J* = 5.6 Hz, 1H, γ-bpy-6), 7.93 (d, *J* = 5.6 Hz, 1H, β-bpy-6), 7.90 (d, *J* = 5.6 Hz, 1H, δ-bpy-6), 7.81–7.79 (m, 5H, α-bpy-6, dmb-6), 7.55 (d, *J* = 5.6 Hz, 1H, β-bpy-5), 7.35–7.27 (m, 17H, α-bpy-5, dmb-5, Ph-*o*), 7.18 (d, *J* = 5.6 Hz, 1H, γ-bpy-5), 7.06 (dd, *J*_HH_ = 7.8 Hz, *J*_FH_ = 7.8 Hz, 12H, Ph-*m*), 6.97 (d, *J* = 5.2 Hz, 1H, δ-bpy-5), 4.86 (s, 2H, β-C*H*_2_), 4.82 (s, 2H, γ-C*H*_2_), 2.54–2.44 (m, 18H, dmb-C*H*_3_ α-bpy-C*H*_3_, δ-bpy-C*H*_3_). FT-IR (in CH_2_Cl_2_) *ν*_CO_/cm^–1^: 1870, 1940. ESI-MS (eluent: MeCN) *m*/*z*: 576 ([M – 3PF_6_^–^]^3+^), 936 ([M – 2PF_6_^–^]^2+^).

The other diad was synthesized in a manner similar to that used to prepare **Ru(CH_2_OCH_2_)Re** but with **Ru(CH_2_SCH_2_)** or **Ru(CH_2_CH_2_CH_2_)** used instead of **Ru(CH_2_OCH_2_)**.

Yield 19%. Elemental analysis calcd (%) for C_86_H_72_F_24_N_8_O_3_P_5_ReRuS: C. 47.04; H. 3.31; N. 5.10; S. 1.46. Found: C. 47.04; H. 3.45; N. 5.09; S. 1.68. ^1^H-NMR (300 MHz, acetone-*d*_6_) *δ*/ppm: 8.76 (s, 1H, γ-bpy-3), 8.66 (s, 5H, β-bpy-3, dmb-3), 8.33 (s, 1H, δ-bpy-3), 8.26 (s, 1H, α-bpy-3), 8.11 (d, *J* = 5.6 Hz, 1H, γ-bpy-6), 7.96 (d, *J* = 5.6 Hz, 1H, β-bpy-6), 7.89–7.81 (m, 6H, α-bpy-6, dmb-6, δ-bpy-6), 7.53 (d, *J* = 6.0 Hz, 1H, β-bpy-5), 7.39–7.29 (m, 17H, α-bpy-5, dmb-5, Ph-*o*), 7.17 (d, *J* = 5.6 Hz, 1H, γ-bpy-5), 7.10 (dd, *J*_HH_ = 8.6 Hz, *J*_FH_ = 8.4 Hz, 12H, Ph-*m*), 6.99 (d, *J* = 5.6 Hz, 1H, δ-bpy-5), 3.94–3.88 (m, 4H, β-C*H*_2_, γ-C*H*_2_), 2.57–2.54 (m, 18H, dmb-C*H*_3_, α-bpy-C*H*_3_, δ-bpy-C*H*_3_). FT-IR (in CH_2_Cl_2_) *ν*_CO_/cm^–1^: 1868, 1939. ESI-MS (eluent: MeCN) *m*/*z*: 581 ([M – 3PF_6_^–^]^3+^), 944 ([M – 2PF_6_^–^]^2+^).

#### [(dmb)_2_Ru(CH_2_CH_2_CH_2_)Re{P(*p*-FPh)_3_}_2_](PF_6_)_3_ (**Ru(CH_2_CH_2_CH_2_)Re**)

Yield 72%. Elemental analysis calcd (%) for C_87_H_72_F_24_N_8_O_2_P_5_ReRu: C. 48.38; H. 3.36; N. 5.19. Found: C. 48.40; H. 3.45; N. 5.06. ^1^H-NMR (300 MHz, acetone-*d*_6_) *δ*/ppm: 8.61 (s, 1H, β-bpy-3), 8.59 (s, 5H, α-bpy-3, dmb-3), 8.19 (s, 1H, γ-bpy-3), 8.16 (s, 1H, δ-bpy-3), 7.98 (d, *J* = 5.3 Hz, 1H, γ-bpy-6), 7.80–7.78 (m, 2H, β-bpy-6, α-bpy-6), 7.74–7.72 (m, 5H, δ-bpy-6, dmb-6), 7.38 (d, *J* = 6.0 Hz, 1H, β-bpy-5), 7.32–7.25 (m, 17H, α-bpy-5, dmb-5, Ph-*o*), 7.04 (dd, *J*_HH_ = 8.6 Hz, *J*_FH_ = 8.6 Hz, 12H, Ph-*m*), 6.96 (d, *J* = 5.6 Hz, 1H, γ-bpy-5), 6.90 (d, *J* = 5.9 Hz, 1H, δ-bpy-5), 2.86 (s, 2H, β-C*H*_2_), 2.78 (s, 2H, γ-C*H*_2_), 2.50–2.40 (m, 18H, dmb-C*H*_3_ α-bpy-C*H*_3_, δ-bpy-C*H*_3_), 2.01 (tt, 4H, *J* = 2.1 Hz, 2.1 Hz, –CH_2_–C*H*_2_–CH_2_–). FT-IR (in CH_2_Cl_2_) *ν*_CO_/cm^–1^: 1868, 1938. ESI-MS (eluent: MeCN) *m*/*z*: 575 ([M – 3PF_6_^–^]^3+^), 935 ([M – 2PF_6_^–^]^2+^).

#### Synthesis of [(dmb)_2_Ru(CH_2_OCH_2_)Re(CO)_3_Br](PF_6_)_2_ (**Ru(CH_2_OCH_2_)Re(CO)_3_Br**)

**Ru(CH_2_OCH_2_)** (68.5 mg, 0.06 mmol) and Re(CO)_5_Br (32.5 mg, 0.08 mmol) were dissolved in EtOH (60 mL), and the solution was refluxed for 5 h under an N_2_ atmosphere in the absence of light. After evaporation of the solvent, **Ru(CH_2_OCH_2_)Re(CO)_3_Br** was isolated by ion-exchange column chromatography on a SP Sephadex C-25 column using acetonitrile–water (1 : 1 v/v) containing NH_4_PF_6_ as the eluent. Although a small amount of **Ru(CH_2_OCH_2_)Re(CO)_3_(MeCN)** byproduct was present as a contaminant, the recovered sample was used in the subsequent synthesis without further purification. Yield: 66.5 mg (crude). FT-IR (in CH_2_Cl_2_) *ν*_CO_/cm^–1^: 2039, 2022, 1919. ESI-MS (eluent: MeCN) *m*/*z*: 600 ([M – 2PF_6_^–^]^2+^).

#### Synthesis of [(dmb)_2_Ru(CH_2_OCH_2_)Re(CO)_3_(MeCN)](PF_6_)_3_·H_2_O (**Ru(CH_2_OCH_2_)Re(CO)_3_(MeCN)**)

The crude sample (75.3 mg) of **Ru(CH_2_OCH_2_)Re(CO)_3_Br** containing **Ru(CH_2_OCH_2_)Re(CO)_3_(MeCN)** and CF_3_SO_3_H (0.03 mL, 0.34 mmol) were dissolved in *o*-dichlorobenzene (7 mL), and the solution was refluxed for 5 h under an Ar atmosphere in the absence of light. After evaporation of the solvent, the product was isolated by ion-exchange column chromatography on a CM Sephadex C-25 column using MeCN–H_2_O (1 : 1 v/v) containing NH_4_PF_6_ as the eluent. After evaporation of the solvent, **Ru(CH_2_OCH_2_)Re(CO)_3_(MeCN)** was obtained by recrystallization from a CH_2_Cl_2_–diethyl ether mixed solution as a red solid. The yield was 70% (56.5 mg, 0.05 mmol). Elemental analysis calcd (%) for C_53_H_51_F_18_N_9_O_5_P_3_ReRu: C, 39.39; H, 3.18; N, 7.80. Found: C, 39.36; H, 3.19; N, 7.71. ^1^H-NMR (300 MHz, acetonitrile-*d*_3_) *δ*/ppm: 8.96 (d, *J* = 5.9 Hz, 1H, β-bpy-6), 8.85 (d, *J* = 5.7 Hz, 1H, α-bpy-6), 8.47 (s, 1H, β-bpy-3), 8.44 (s, 1H, α-bpy-3), 8.37 (s, 1H, γ-bpy-3), 8.34 (s, 1H, δ-bpy-3), 8.32 (s, 4H, dmb-3), 7.70 (d, *J* = 5.7 Hz, 1H, γ-bpy-6), 7.68 (d, *J* = 6.2 Hz, 1H, β-bpy-5), 7.55–7.49 (m, 6H, δ-bpy-6, dmb-6, α-bpy-5), 7.39 (d, *J* = 5.7 Hz, 1H, γ-bpy-5), 7.21–7.20 (m, 5H, δ-bpy-5, dmb-5), 4.91 (s, 2H, β-CH_2_), 4.88 (s, 2H, γ-CH_2_), 2.56–2.51 (m, 18H, dmb-CH_3_, β-bpy-CH_3_, γ-bpy-CH_3_), 2.04 (s, 3H, CH_3_–CN–). FT-IR (in MeCN) *ν*_CO_/cm^–1^: 2039, 1934. ESI-MS (eluent: MeCN) *m*/*z*: 600 ([M – MeCN + Br^–^ – 3PF_6_^–^]^3+^), 655 ([M – 2PF_6_^–^]^2+^).

#### Synthesis of [(dmb)_2_Ru(CH_2_OCH_2_)Re(CO)_3_(OC(O)OC_2_H_4_N(C_2_H_4_OH)_2_)](PF_6_)_2_ (**Ru(CH_2_OCH_2_)Re(CO)_3_(X)**)

**Ru(CH_2_OCH_2_)Re(CO)_3_(MeCN)** was dissolved in DMF, and the resulting solution was stirred overnight. TEOA was added to the solution, and the solution was stirred for an hour to give a mixture of **Ru(CH_2_OCH_2_)Re(CO)_3_(DMF)** and **Ru(CH_2_OCH_2_)Re(CO)_3_(TEOA)**. **Ru(CH_2_OCH_2_)Re(CO)_3_(X)** was obtained by bubbling the solution with CO_2_ for 10 min. The reversibility of this CO_2_ insertion reaction was confirmed by the recovery of the starting complexes in the solution after bubbling with Ar for 20 min. FT-IR (in MeCN) *ν*_CO_/cm^–1^: 2006, 2018 (Fig. S5, ESI†).

## Conclusions

The greatest photocatalytic activity for CO_2_ reduction using BNAH as the reductant was achieved using **Ru(CH_2_OCH_2_)Re** (*Φ*_CO_ = 0.18, TON_CO_ = 253). Introduction of an oxygen atom into the bridging unit improved the oxidation power of the excited Ru unit in the diad and increased the driving force of the intramolecular electron transfer from the OER species of the Ru unit to the Re unit. During the photocatalytic reaction, a part of the used **Ru(CH_2_OCH_2_)Re** was converted into **Ru(CH_2_OCH_2_)Re(CO)_3_(X)** (X = ^–^OC_2_H_4_N(C_2_H_4_OH)_2_ or ^–^OC(O)OC_2_H_4_N(C_2_H_4_OH)_2_) during the initial stage of the photocatalytic reaction. The 3 : 1 mixture of **Ru(CH_2_OCH_2_)Re** and **Ru(CH_2_OCH_2_)Re(CO)_3_(X)**, which respectively functioned as a photosensitizer and a photocatalyst in the photostationary state, efficiently photocatalyzed CO_2_ reduction for an extended period.

## Supplementary Material

Supplementary information
