# Supplementary material for: Ru(ii)–Re(i) binuclear photocatalysts connected by –CH2XCH2– (X = O, S, CH2) for CO2 reduction
Source: Chem Sci. 2015 Mar 2;6(5):3003–12. doi: 10.1039/c4sc03710c (PMC5729417; doi:10.1039/c4sc03710c)
Supplement: Supplementary file 1 [file SC-006-C4SC03710C-s001.pdf]

Electronic Supplementary Material (ESI) for Chemical Science.
This journal is © The Royal Society of Chemistry 2015

Ru(II)-Re(I) Binuclear Photocatalysts Connected by $–CH_2XCH_2–$ ($X = O, S, CH_2$) for CO_2 Reduction

Eishiro Kato,[a] Hiroyuki Takeda,[a,b] Kazuhide Koike,[b,c] Kei Ohkubo,[a] and Osamu Ishitani[a,b*]

Supporting Information

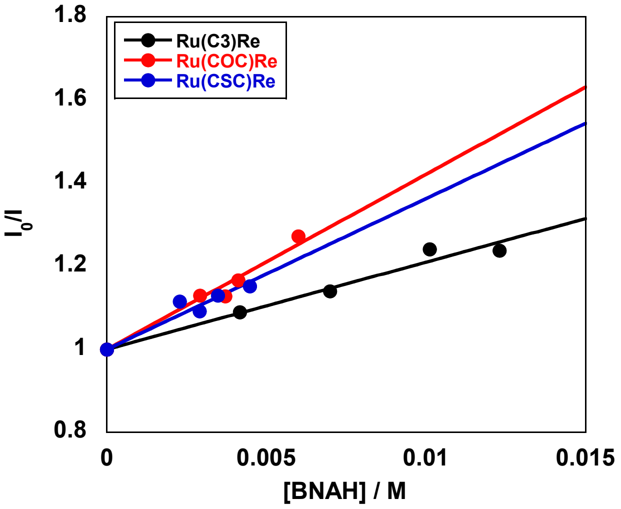

Figure S1. The Stern-Volmer plot of the diads.

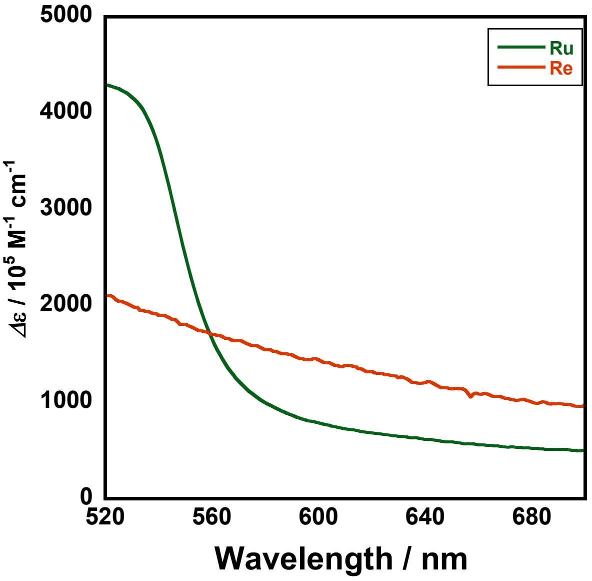

Figure S2. UV-vis absorption spectra of OER species in an Ar-saturated CH_3CN solution containing 0.1 M Et_4NBF_4 at room temperature obtained by flow electrolysis method: **Ru** (green line) and **Re** (orange line).[S1]

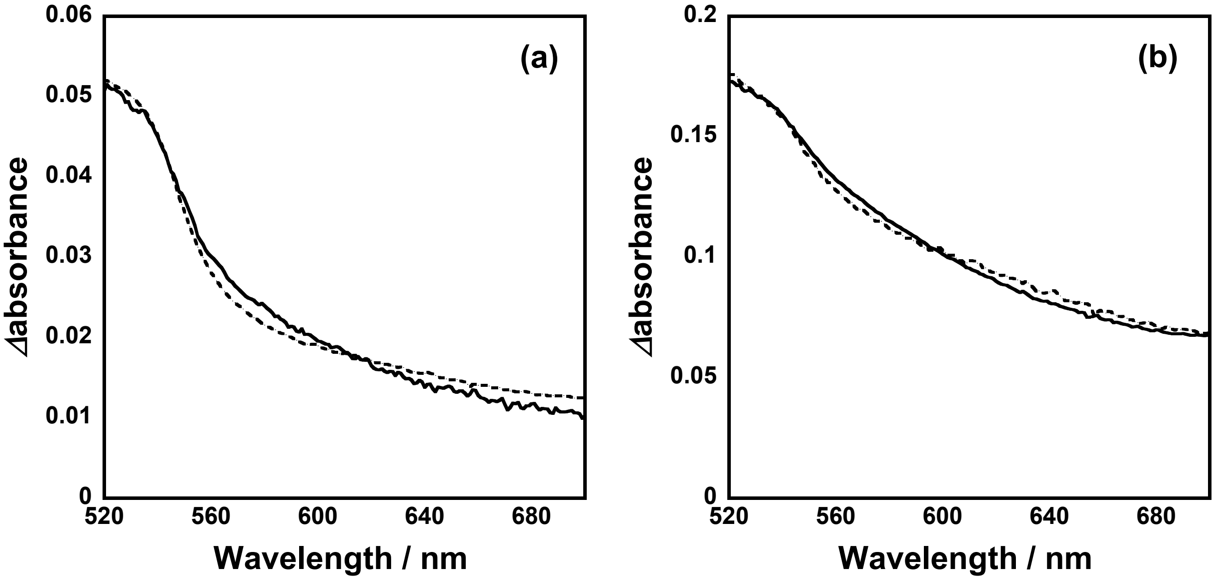

Figure S3. Differential UV-vis absorption spectrum of the photocatalytic reaction solution using (a): **Ru($CH_2CH_2CH_2$)Re** before and after irradiation for 1080 s (solid line); (b): **Ru(CH_2OCH_2)Re** before and after irradiation for 2610 s (solid line) and the simulated spectrum using the spectra of the OER species of **Ru** and **Re** (broken line).

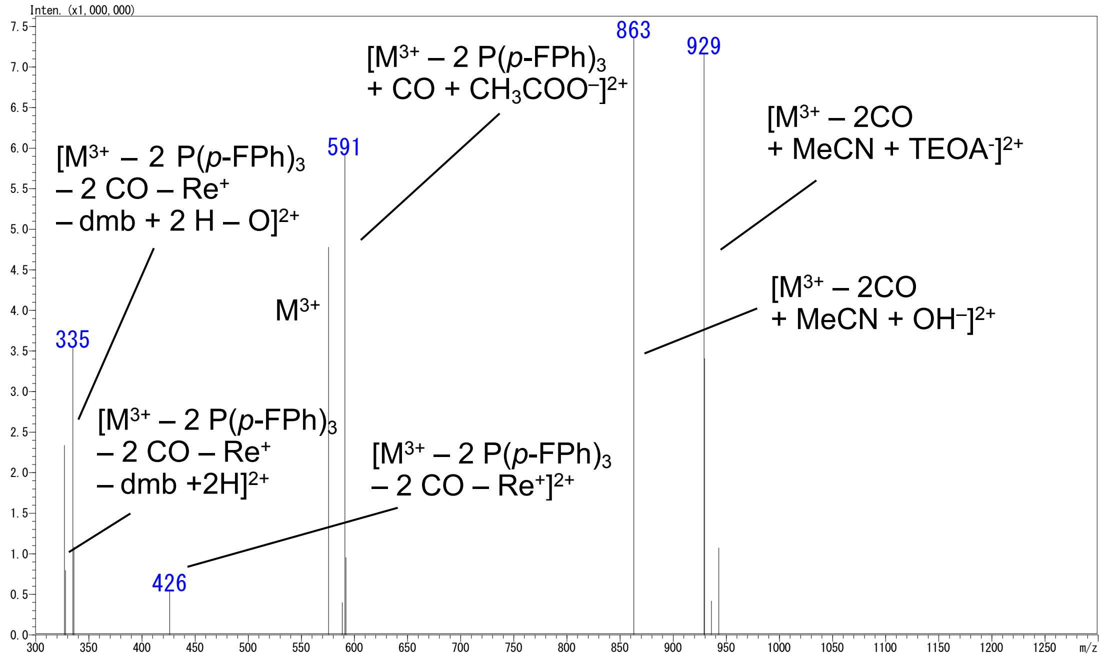

Figure S4. ESI-MS spectrum of the eluate at 36.1 min of retention time separated from the reaction solution after the photocatalytic reaction (120 min photoirradiation) of **Ru(CH_2OCH_2)Re** (M^{3+}) by SEC using an MeCN-MeOH (1:1 v/v) mixed eluent containing 0.05 M $NH_4^+CH_3COO^-$ with a shodex PROTEIN KW402.5 columns and a KW-LG guard-column under the following conditions: flow rate, 0.2 ml min^{-1}; column temperature, 40 ^oC.

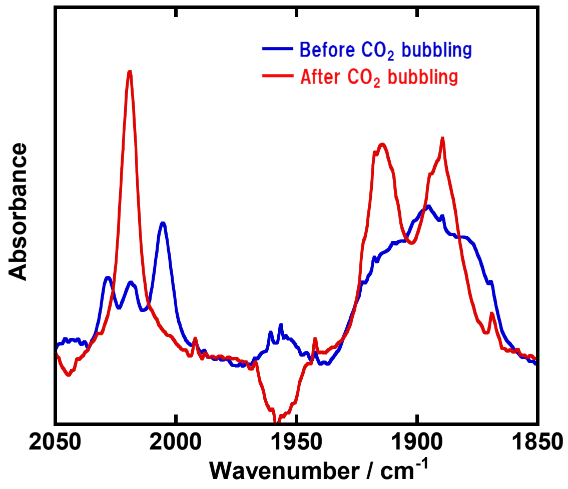

Figure S5. IR spectra of a DMF-TEOA (5:1 v/v) mixed solution containing **Ru(CH_2OCH_2)Re(X)** (**X** = DMF or TEOA) before and after CO_2 bubbling for 15 min.

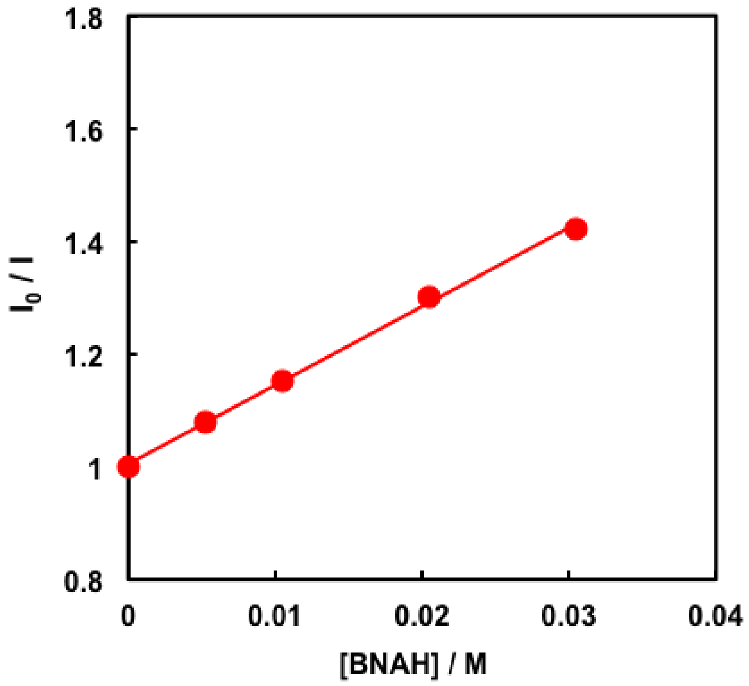

Figure S6. The Stern-Volmer plot of **Ru(CH_2OCH_2)Re($(CO)_3$)(X)** (X = $^-OC(O)OC_2H_4N(C_2H_4OH)_2$).

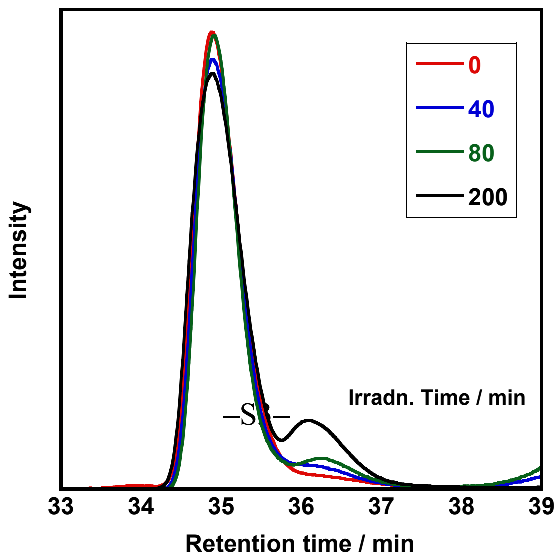

Figure S7. Analytical-SEC chromatograms of the solution after the photocatalytic reaction (irradiation time: 0, 40, 80 and 200 min) with a shodex PROTEIN KW402.5 columns and a KW-LG guard-column using an MeCN-MeOH (1:1 v/v) mixed eluent containing 0.5 M $NH_4^+CH_3COO^-$ under the following conditions: flow rate, 0.2 ml min^{-1}; column temperature, 40 °C; detection wavelength, 390 nm. In the photocatalytic reaction, CO_2 saturated DMF–TEOA solutions containing BNAH (0.1 M) and **Ru($CH_2CH_2CH_2$)Re** (0.3 mM) were irradiated at 480 nm using the lamp house apparatus with light intensity of 3.2×10^{-9} einstein s^{-1}.

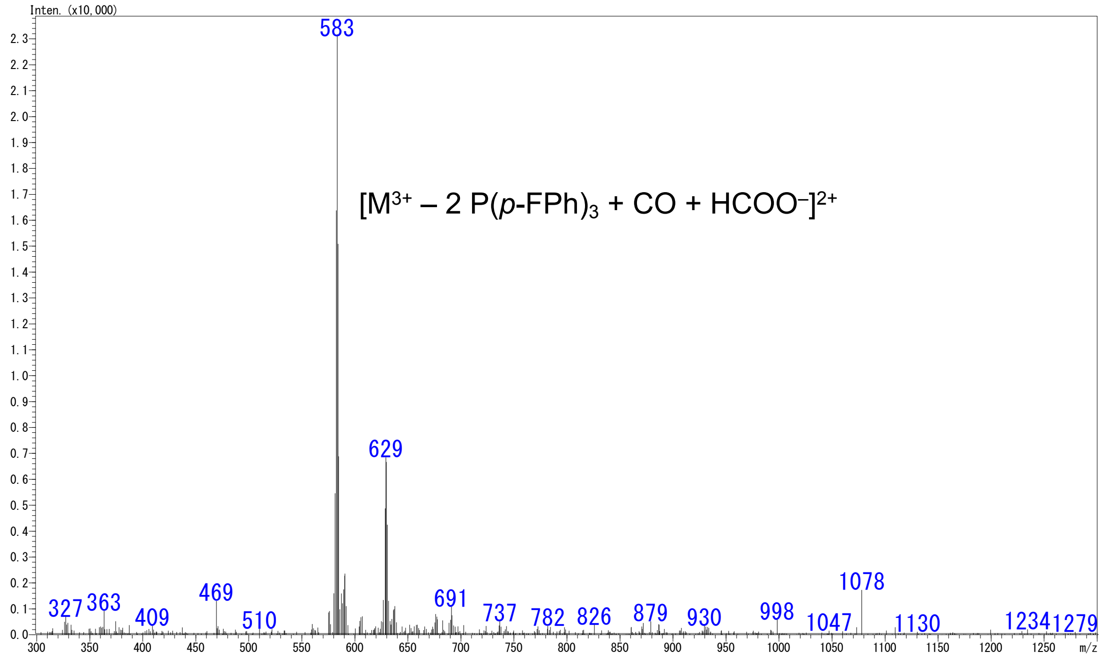

Figure S8. ESI-MS spectrum of the eluate at 36.1 min of retention time separated from the reaction solution after the photocatalytic reaction (120 min photoirradiation) of **Ru($CH_2CH_2CH_2$)Re** (M^{3+}) by SEC using an MeCN-MeOH (1:1 v/v) mixed eluent containing 0.05 M $NH_4^+CH_3COO^-$ with a shodex PROTEIN KW402.5 columns and a KW-LG guard-column under the following conditions: flow rate, 0.2 ml min^{-1}; column temperature, 40 °C.

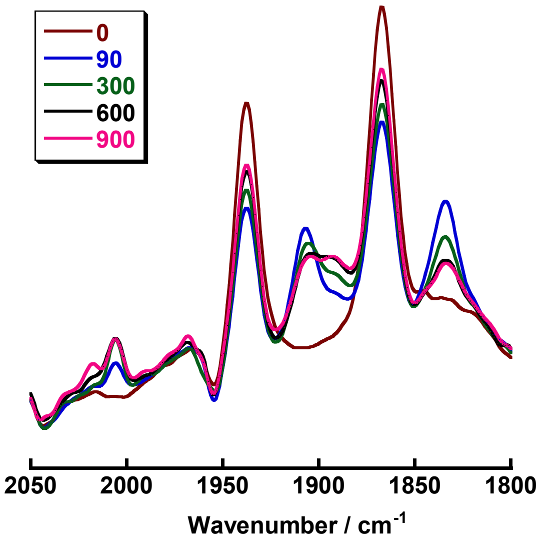

Figure S9. *In-situ* IR spectra of a DMF-TEOA (5:1 v/v) solutions containing **Ru($CH_2CH_2CH_2$)Re** (1 mM) and BNAH (0.1 M) during photoirradiation using 480-nm light under a CO_2 atmosphere.

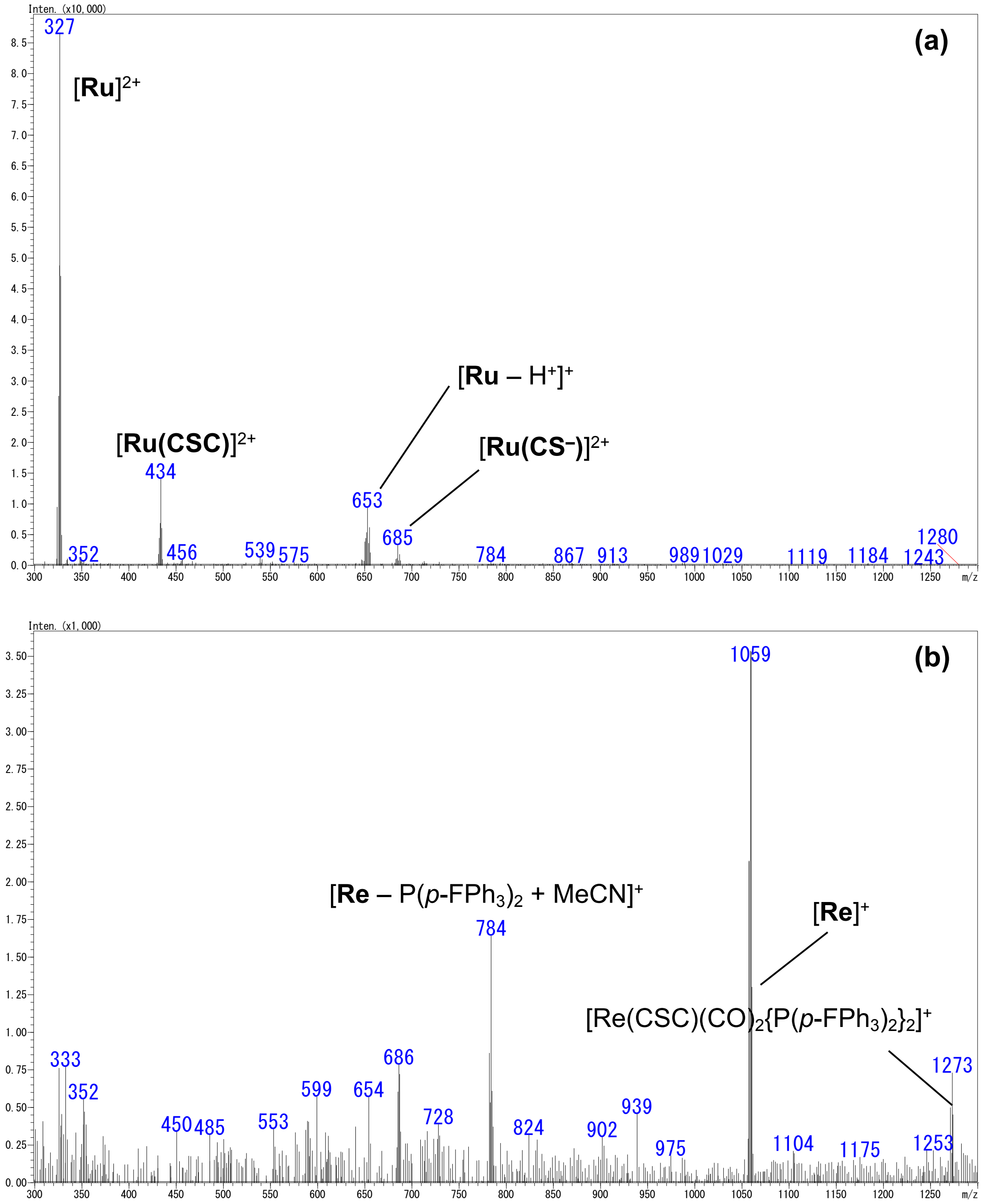

Figure S10. ESI-MS spectrum of the eluates at 36.5 min (a) and 37.8 min (b) of retention time separated from the reaction solution after the photocatalytic reaction (120 min photoirradiation) of **Ru(CH$_2$SCH$_2$)Re** (M^{3+}) by SEC using an MeCN-MeOH (1:1 v/v) mixed eluent containing 0.05 M $NH_4^+CH_3COO^-$ with a shodex PROTEIN KW402.5 columns and a KW-LG guard-column under the following conditions: flow rate, 0.2 ml min^{-1}; column temperature, 40 ^{o}C.

Reference

S1. H. Tsubaki, A. Sugawara, H. Takeda, B. Gholamkhass, K. Koike and O. Ishitani, *Res. Chem. Intermed.*, 2007, **33**, 37.
